# Influence of Fatty Infiltration of Muscle on Falls and Fall-Related Outcomes in Middle-Aged and Older Adults: A Systematic Review

**DOI:** 10.7759/cureus.82345

**Published:** 2025-04-16

**Authors:** Atsushi Shinonaga, Hiromi Matsumoto, Kensuke Tochio, Shigeharu Tanaka, Naoki Deguchi, Ryo Tanaka

**Affiliations:** 1 Graduate School of Humanities and Social Sciences, Hiroshima University, Higashihiroshima, JPN; 2 Rehabilitation Center, Kawasaki Geriatric Medical Center, Okayama, JPN; 3 Physical Therapy, Faculty of Rehabilitation, Kawasaki University of Medical Welfare, Kurashiki, JPN; 4 Rehabilitation, Faculty of Health Sciences, Tokyo Kasei University, Sayama, JPN; 5 Geriatrics, Tokyo Metropolitan Institute for Geriatrics and Gerontology, Tokyo, JPN

**Keywords:** fall-related outcomes, falls, fatty infiltration of muscle, middle-aged, older adults

## Abstract

Fatty infiltration of muscle is increasingly recognized as a factor contributing to fall risk in middle-aged and older adults. The goal of this study is to systematically review the literature on the influence of fatty infiltration of muscle on falls and fall-related outcomes in middle-aged and older adults. Five databases - PubMed, the Cochrane Central Register of Controlled Trials, Cumulative Index to Nursing and Allied Health Literature, Web of Science, and Igaku Chuo Zasshi - were comprehensively searched. In addition, relevant studies were identified through hand searching of the reference lists of included articles. Study quality was assessed using the Newcastle-Ottawa scale. We initially identified 1,450 articles through database searches and an additional 26 articles through hand searching. After screening, 97 observational studies were included in the final analysis. Medical imaging modalities for fatty infiltration of muscle were CT, MRI, ultrasonography, and peripheral quantitative CT. Outcomes included falls, comfortable walking speed, maximum walking speed, the timed up and go test, the short physical performance battery, muscle strength, the 6-min walk test, the five times sit-to-stand test, the 30-s chair stand test, and other balance tests. Most of the surveyed studies indicated a potential association between lower extremity major fatty infiltration of muscle and falls. Similarly, fatty infiltration of muscle was associated with poorer fall-related outcomes. However, these studies included varied participant characteristics and methods for assessing fatty infiltration of muscle and exhibited a mixed risk of bias. In conclusion, our systematic review provides important evidence on assessing fatty infiltration of the muscle for fall prevention in older adults, while underscoring the need for careful interpretation and further research, considering variations in participant characteristics, assessment methods, and potential biases.

## Introduction and background

Falls among older adults are a notable global public health concern. According to the 2019 Global Burden of Disease study, falls are the second-leading cause of accidental injury-related deaths among individuals aged 50-74, exceeded only by road traffic injuries [1]. They are also associated with fractures [2], reduced activity [3], nursing home admissions [4], and anxiety disorders [5], collectively increasing the burden on caregivers and healthcare costs [6]. Identifying fall risk factors in older adults and implementing appropriate interventions are therefore vital to extending healthy life expectancy and controlling healthcare expenditures.

A decline in skeletal muscle mass in older adults is a key contributor to fall risk. Decreased total body skeletal muscle mass is closely linked to past falls and an elevated risk of future falls [7,8]. Moreover, the lower quadriceps muscle mass, which is crucial for mobility, further increases fall risk [9]. Consequently, evaluating both total and site-specific skeletal muscle masses in older adults is crucial for effective fall prevention.

Over the past decade, knowledge has been growing not only about muscle mass but also about muscle quality. Muscle quality is generally defined as muscle strength or power per unit of muscle mass [10]. It is influenced by various morphological changes in muscles, such as fat infiltration, muscle composition (e.g., architecture and fiber type), metabolism, fibrosis, and neural activation [11]. A recent scoping review has emphasized the value of assessing muscle quality - particularly its morphological aspects - as a useful indicator of physical function in older adults [12]. Several longitudinal studies have found that muscle mass deterioration alone does not fully account for reductions in muscle strength and physical function, suggesting that muscle morphology changes play a critical role [13-15]. These changes may contribute to falls by impairing contractile function and diminishing essential physical abilities such as walking speed and dynamic balance. However, despite growing interest, the current evidence linking muscle morphology changes to fall risk remains limited, fragmented, and inconclusive. This ambiguity hampers the development of effective preventive strategies. Therefore, further investigation is needed to clarify the impact of muscle morphology changes on fall risk in aging populations.

In this study, we focus on fatty infiltration as a key indicator of morphological muscle quality. Fatty infiltration of muscle refers to the accumulation of adipose tissue within and between skeletal muscles [11]. Fatty infiltration of muscle measurability using imaging techniques such as CT, MRI, and ultrasonography makes it a practical and reliable parameter in clinical and community settings [11]. Moreover, the propensity for age-related fatty infiltration occurring at an earlier stage than muscle mass decline underscores the importance of interventions from middle age onward [16]. Thus, fatty infiltration of muscle assessment may be crucial for identifying older adults at high risk of falls and for preventing midlife-related declines in physical function associated with fall risk. Although the relationship between fatty infiltration of muscle and falls has been examined in several previous studies, a unified finding has not been reached and the evidence is limited. Clarifying the extent to which this relatively new measure, fatty infiltration of muscle, affects falls could provide new clinical intervention on fall prevention in clinical practice. The goal of this study is to systematically review the literature on the influence of fatty infiltration of muscle on falls and fall-related outcomes in middle-aged and older adults.

## Review

Methods

This systematic review was performed according to the Preferred Reporting Items for Systematic reviews and Meta-Analyses (PRISMA) guidelines 2020 [17]. The protocol is available on the University Hospital Medical Information Network (UMIN000054584).

Data Sources and Search Strategy

A systematic search was undertaken on November 10, 2024 using the following databases: PubMed, the Cochrane Central Register of Controlled Trials, Cumulative Index to Nursing and Allied Health Literature, Web of Science, and Igaku Chuo Zasshi (a Japanese database). Igaku Chuo Zasshi, the largest medical database in Japan, was included in this study to minimize language bias. The terms for exposure factors included “fatty infiltration of muscle” and “intramuscular adipose tissue”. Outcome-related terms included “falls”, “physical function”, “gait”, “muscle strength”, and “balance”. The study designs included “cohort study”, “cross-sectional study”, and “case-control study”. An example of the search strategy used in the PubMed database is shown in Table 1. In addition, relevant studies were identified through hand searching of the reference lists of included articles.

**Table 1:** Search strategy

Search		Query
Exposure AND Outcomes AND Study design	#14	Search: #1 AND #10 AND #13
Study design	#13	Search: #11 OR #12
	#12	Search: ((((((((((((((((((((((((((((((("cross-sectional study"[Title/Abstract]) OR ("Cross-Sectional Analysis"[Title/Abstract])) OR ("Cross-Sectional Study"[Title/Abstract])) OR ("Cross-Sectional Survey"[Title/Abstract])) OR ("Disease Frequency Survey"[Title/Abstract])) OR ("Prevalence Study"[Title/Abstract])) OR ("Cross-Sectional Studies"[MeSH Terms])) OR ("Cross Sectional Studies"[Title/Abstract])) OR ("Cross-Sectional Study"[Title/Abstract])) OR ("Studies, Cross-Sectional"[Title/Abstract])) OR ("Study, Cross-Sectional"[Title/Abstract])) OR ("Cross Sectional Analysis"[Title/Abstract])) OR ("Analyses, Cross Sectional"[Title/Abstract])) OR ("Cross Sectional Analyses"[Title/Abstract])) OR ("Disease Frequency Surveys"[Title/Abstract])) OR ("Cross-Sectional Survey"[Title/Abstract])) OR ("Cross Sectional Survey"[Title/Abstract])) OR ("Cross-Sectional Surveys"[Title/Abstract])) OR ("Survey, Cross-Sectional"[Title/Abstract])) OR ("Surveys, Cross-Sectional"[Title/Abstract])) OR ("Surveys, Disease Frequency"[Title/Abstract])) OR ("Disease Frequency Survey"[Title/Abstract])) OR ("Survey, Disease Frequency"[Title/Abstract])) OR ("Analysis, Cross-Sectional"[Title/Abstract])) OR ("Analyses, Cross-Sectional"[Title/Abstract])) OR ("Analysis, Cross Sectional"[Title/Abstract])) OR ("Cross-Sectional Analyses"[Title/Abstract])) OR ("Cross-Sectional Analysis"[Title/Abstract])) OR ("Prevalence Studies"[Title/Abstract])) OR ("Prevalence Study"[Title/Abstract])) OR ("Studies, Prevalence"[Title/Abstract])) OR ("Study, Prevalence"[Title/Abstract])
	#11	Search: (((((((((((((((((((((((((((((((((((((((((((((((((((((((((((((((((((((((((((((((((((((((((((((((((((((("cohort study"[Title/Abstract]) OR ("Closed Cohort Study"[Title/Abstract])) OR (Cohort Analysis[Title/Abstract])) OR (Cohort Study[Title/Abstract])) OR (Concurrent Study[Title/Abstract])) OR (Historical Cohort Study[Title/Abstract])) OR (Incidence Study[Title/Abstract])) OR (Cohort Studies[MeSH Terms])) OR (Cohort Study[Title/Abstract])) OR (Studies, Cohort[Title/Abstract])) OR (Study, Cohort[Title/Abstract])) OR (Concurrent Studies[Title/Abstract])) OR (Studies, Concurrent[Title/Abstract])) OR (Concurrent Study[Title/Abstract])) OR (Study, Concurrent[Title/Abstract])) OR (Closed Cohort Studies[Title/Abstract])) OR (Cohort Studies, Closed[Title/Abstract])) OR (Closed Cohort Study[Title/Abstract])) OR (Cohort Study, Closed[Title/Abstract])) OR (Study, Closed Cohort[Title/Abstract])) OR (Studies, Closed Cohort[Title/Abstract])) OR (Birth Cohort Studies[Title/Abstract])) OR (Birth Cohort Study[Title/Abstract])) OR (Cohort Studies, Birth[Title/Abstract])) OR (Cohort Study, Birth[Title/Abstract])) OR (Studies, Birth Cohort[Title/Abstract])) OR (Study, Birth Cohort[Title/Abstract])) OR (Analysis, Cohort[Title/Abstract])) OR (Analyses, Cohort[Title/Abstract])) OR (Cohort Analyses[Title/Abstract])) OR (Cohort Analysis[Title/Abstract])) OR (Historical Cohort Studies[Title/Abstract])) OR (Cohort Studies, Historical[Title/Abstract])) OR (Cohort Study, Historical[Title/Abstract])) OR (Historical Cohort Study[Title/Abstract])) OR (Study, Historical Cohort[Title/Abstract])) OR (Studies, Historical Cohort[Title/Abstract])) OR (Incidence Studies[Title/Abstract])) OR (Incidence Study[Title/Abstract])) OR (Studies, Incidence[Title/Abstract])) OR (Study, Incidence[Title/Abstract])) OR (case-control study[Title/Abstract])) OR ("Case-Base Study"[Title/Abstract])) OR ("Case-Comparison Study"[Title/Abstract])) OR ("Case-Compeer Study"[Title/Abstract])) OR ("Case-Control Study"[Title/Abstract])) OR ("Case-Referent Study"[Title/Abstract])) OR ("Matched Case-Control Study"[Title/Abstract])) OR ("Nested Case-Control Study"[Title/Abstract])) OR ("Case-Control Studies"[MeSH Terms])) OR ("Case-Control Study"[Title/Abstract])) OR ("Studies, Case-Control"[Title/Abstract])) OR ("Study, Case-Control"[Title/Abstract])) OR ("Case-Comparison Studies"[Title/Abstract])) OR ("Case Comparison Studies"[Title/Abstract])) OR ("Case-Comparison Study"[Title/Abstract])) OR ("Studies, Case-Comparison"[Title/Abstract])) OR ("Study, Case-Comparison"[Title/Abstract])) OR ("Case-Compeer Studies"[Title/Abstract])) OR ("Studies, Case-Compeer"[Title/Abstract])) OR ("Case-Referent Studies"[Title/Abstract])) OR ("Case Referent Studies"[Title/Abstract])) OR ("Case-Referent Study"[Title/Abstract])) OR ("Studies, Case-Referent"[Title/Abstract])) OR ("Study, Case-Referent"[Title/Abstract])) OR ("Case-Base Studies"[Title/Abstract])) OR ("Case Base Studies"[Title/Abstract])) OR ("Studies, Case-Base"[Title/Abstract])) OR ("Case Control Studies"[Title/Abstract])) OR ("Case Control Study"[Title/Abstract])) OR ("Studies, Case Control"[Title/Abstract])) OR ("Study, Case Control"[Title/Abstract])) OR ("Nested Case-Control Studies"[Title/Abstract])) OR ("Case-Control Studies, Nested"[Title/Abstract])) OR ("Case-Control Study, Nested"[Title/Abstract])) OR ("Nested Case Control Studies"[Title/Abstract])) OR ("Nested Case-Control Study"[Title/Abstract])) OR ("Studies, Nested Case-Control"[Title/Abstract])) OR ("Study, Nested Case-Control"[Title/Abstract])) OR ("Matched Case-Control Studies"[Title/Abstract])) OR ("Case-Control Studies, Matched"[Title/Abstract])) OR ("Case-Control Study, Matched"[Title/Abstract])) OR ("Matched Case Control Studies"[Title/Abstract])) OR ("Matched Case-Control Study"[Title/Abstract])) OR ("Studies, Matched Case-Control"[Title/Abstract])) OR ("Study, Matched Case-Control"[Title/Abstract])) OR ("prospective study"[Title/Abstract])) OR ("prospective analysis"[Title/Abstract])) OR ("prospective cohort study"[Title/Abstract])) OR ("prospective evaluation"[Title/Abstract])) OR ("prospective randomized trial"[Title/Abstract])) OR ("Prospective Study"[Title/Abstract])) OR ("Prospective Studies"[MeSH Terms])) OR ("Prospective Study"[Title/Abstract])) OR ("Studies, Prospective"[Title/Abstract])) OR ("Study, Prospective"[Title/Abstract])) OR ("retrospective study"[Title/Abstract])) OR ("retrospective cohort study"[Title/Abstract])) OR ("Retrospective Study"[Title/Abstract])) OR ("Retrospective Studies"[MeSH Terms])) OR ("Studies, Retrospective"[Title/Abstract])) OR ("Study, Retrospective"[Title/Abstract])) OR ("Retrospective Study"[Title/Abstract])
Outcomes	#10	Search: #2 OR #3 OR #4 OR #5 OR #6 OR #7 OR #8OR #9
	#9	Search: ((((((((((((("mobility"[Title/Abstract]) OR ("Mobility Limitation"[MeSH Terms])) OR ("Limitation, Mobility"[Title/Abstract])) OR ("Mobility Limitations"[Title/Abstract])) OR ("Ambulation Difficulty"[Title/Abstract])) OR ("Ambulation Difficulties"[Title/Abstract])) OR ("Difficulties, Ambulation"[Title/Abstract])) OR ("Difficulty, Ambulation"[Title/Abstract])) OR ("Difficulty Ambulation"[Title/Abstract])) OR ("Ambulatory Difficulty"[Title/Abstract])) OR ("Ambulatory Difficulties"[Title/Abstract])) OR ("Difficulties, Ambulatory"[Title/Abstract])) OR ("Difficulty Walking"[Title/Abstract])) OR ("Walking, Difficulty"[Title/Abstract])
	#8	Search: ((((((((("muscle strength"[Title/Abstract]) OR ("arthrogenic muscle inhibition"[Title/Abstract])) OR ("muscle force"[Title/Abstract])) OR ("muscular power"[Title/Abstract])) OR ("muscle strength"[MeSH Terms])) OR ("strength, muscle"[Title/Abstract])) OR ("arthrogenic muscle inhibition"[Title/Abstract])) OR ("arthrogenic muscle inhibitions"[Title/Abstract])) OR ("inhibition, arthrogenic muscle"[Title/Abstract])) OR ("muscle inhibition, arthrogenic"[Title/Abstract])
	#7	Search: "balance"[Title/Abstract]
	#6	Search: (((((((((((((((((((((((("gait"[Title/Abstract]) OR ("cadence"[Title/Abstract])) OR ("pace"[Title/Abstract])) OR (gait[MeSH Terms])) OR ("gaits"[Title/Abstract])) OR ("walking"[Title/Abstract])) OR ("ambulation"[Title/Abstract])) OR ("locomotor"[Title/Abstract])) OR (walking[MeSH Terms]))
	#5	Search: "motor function"[Title/Abstract]
	#4	Search: ((((((((((((("physical functional performance"[MeSH Terms]) OR ("functional performance, physical"[Title/Abstract])) OR ("functional performances, physical"[Title/Abstract])) OR ("performance, physical functional"[Title/Abstract])) OR ("performances, physical functional"[Title/Abstract])) OR ("physical functional performances"[Title/Abstract])) OR ("functional performance"[Title/Abstract])) OR ("functional performances"[Title/Abstract])) OR ("performance, functional"[Title/Abstract])) OR ("performances, functional"[Title/Abstract])) OR ("physical performance"[Title/Abstract])) OR ("performance, physical"[Title/Abstract])) OR ("performances, physical"[Title/Abstract])) OR ("physical performances"[Title/Abstract])
	#3	Search: "physical function"[Title/Abstract]
	#2	Search: (((((((((((("fall"[Title/Abstract]) OR "slip"[Title/Abstract])) OR ("accidental falls"[MeSH Terms])) OR ("falls"[Title/Abstract])) OR ("falling"[Title/Abstract])) OR ("falls, accidental"[Title/Abstract])) OR ("accidental fall"[Title/Abstract])) OR ("fall, accidental"[Title/Abstract])) OR ("faller"[Title/Abstract])) OR ("fallers"[Title/Abstract])) OR ("tripping"[Title/Abstract])
Exposure	#1	Search: (((((((((((((("muscle quality"[Title/Abstract]) OR ("muscle mass and quality"[Title/Abstract])OR ("specific tension"[Title/Abstract])) OR ("echo intensity"[Title/Abstract])) OR ("echogenicity"[Title/Abstract])) OR ("intermuscular adipose tissue"[Title/Abstract])) OR ("intermuscular fat"[Title/Abstract])) OR ("intramuscular adipose tissue"[Title/Abstract])) OR ("intramuscular fat"[Title/Abstract])) OR ("muscle attenuation"[Title/Abstract])) OR ("muscle density"[Title/Abstract])) OR ("radiological density"[Title/Abstract])) OR ("muscle composition"[Title/Abstract])) OR ("muscle fat infiltration"[Title/Abstract])) OR ("fatty infiltration"[Title/Abstract])) OR ("myosteatosis"[Title/Abstract])) OR ("muscle architecture"[Title/Abstract])) OR ("radiodensity"[Title/Abstract])) OR ("skeletal Muscle radiodensity"[Title/Abstract]))

Eligibility Criteria

Studies were included in this systematic review based on the following criteria: (1) participants aged ≥40 years old; (2) fatty infiltration of muscle was quantitatively assessed; (3) either the incidence of falls was investigated or fall-related outcomes (e.g., gait speed, balance, and muscle strength) were quantitatively evaluated; (4) the study design was an observational study (e.g., cross-sectional, cohort, or case-control studies); (5) the study was an original research article; and (6) the publication was written in English or Japanese. Exclusion criteria were as follows: (1) participants had neuromuscular disease; (2) participants had undergone lower limb amputation; or (3) the outcomes were not falls or fall-related outcomes.

Study Selection

The titles and abstracts of all papers were independently assessed for eligibility by two (AS and KT) reviewers using predefined inclusion and exclusion criteria. Papers that could not be definitively excluded based on their titles or abstracts were retained for further evaluation. For papers that advanced to full-text review, two reviewers (AS and KT) independently assessed their eligibility for inclusion. If both reviewers agreed that a paper met the criteria, it was included in the data extraction process. Any disagreements during the screening or selection process were resolved through discussion, and a third co-author (RT) was consulted when necessary to achieve consensus.

Data Extraction

The following outcomes and variables were extracted independently by two reviewers (AS and KT) from the included studies: outcomes (falls and fall-related outcomes), author, year of publication, study population, study design, sample size, mean or median age of participants, percentage of females, methods used to evaluate exposure factors (e.g., medical imaging modalities, measured indicators, and target muscle), and main results. All extracted data were managed using a Microsoft Excel spreadsheet.

Quality Assessment

The risk of bias in the included studies was independently assessed by two reviewers (AS and KT) using the Newcastle-Ottawa Scale (NOS) [18,19] for cohort and case-control studies and the modified NOS [20,21] for cross-sectional studies. The NOS evaluates three domains: selection, comparability, and outcomes. For cohort and case-control studies, the NOS assigns scores ranging from 0 to 9 points, with studies scoring 7 or more points classified as high quality [18]. For cross-sectional studies, the modified NOS assigns scores ranging from 0 to 7 points, and studies scoring 4 or more points are considered to be of high quality, based on previous research [20].

Results

Study Selection

**Figure 1:**
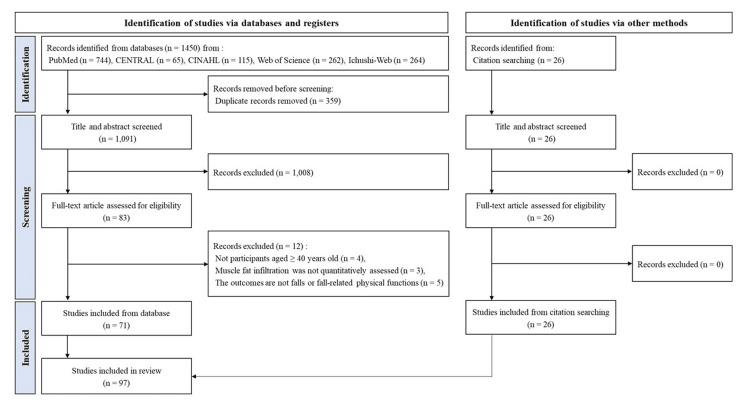
PRISMA flow diagram PRISMA, Preferred Reporting Items for Systematic reviews and Meta-Analyses

The detailed process of literature searching and study selection was presented in the flow chart (Figure 1). We initially identified 1,450 articles through database searches and an additional 26 articles through hand searching. Eventually, 97 eligible articles were included in the systematic review.

Study Characteristics

The medical imaging modalities employed to evaluate fatty infiltration of muscle were CT [13,14,22-55], MRI [56-70], ultrasonography [71-108], and peripheral quantitative CT (pQCT) [109-116]. CT and pQCT have used muscle density as an indicator for fatty infiltration of muscle [13,14,22-25,27-31,33-35,37,41-50,52-54,109-116]. Lower muscle density indicates more advanced inter- and intramuscular fatty infiltration [12]. In addition, CT-based methods have employed specific CT value thresholds to quantify adipose tissue, thereby measuring fat content per unit of muscle cross-sectional area [26,32,36,38-40,51,55]. MRI assessments have used a pixel-intensity histogram within a region of interest to differentiate fat from muscle tissue [56-65,67-70]. Ultrasonography evaluations have utilized echo intensity as a fatty infiltration of muscle index [71-108]. Higher echo intensity indicates more advanced inter- and intramuscular fatty infiltration [12]. Fall-related outcomes included comfortable walking speed, maximum walking speed, the timed up and go test (TUG), the short physical performance battery (SPPB), muscle strength, the 6-min walk test (6MWT), the five times sit-to-stand test (5STS), the 30-s chair stand test (CS-30), and other balance tests. Some studies assessed multiple outcomes concurrently.

Association between fatty infiltration of muscle and incidence of falls

**Figure 2:**
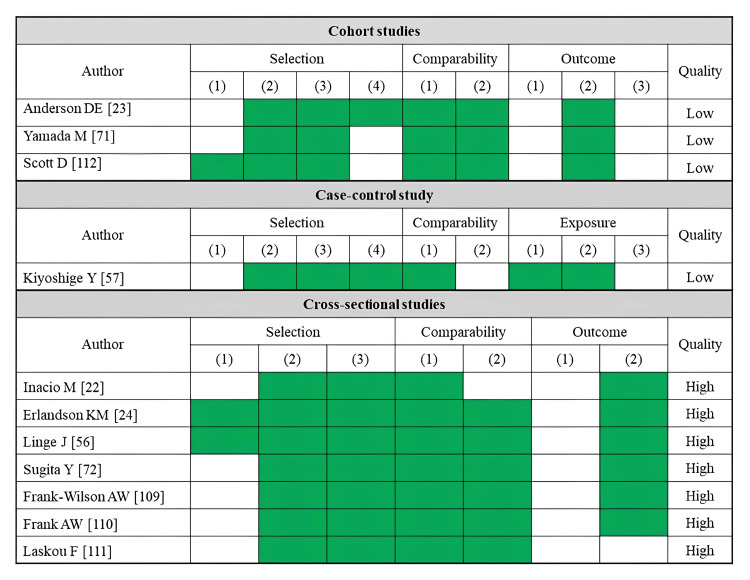
Assessment of risk of bias in studies on falls

A total of 11 studies reported falls as an outcome (Table 2). Fatty infiltration of muscle was assessed by CT (three studies), MRI (two studies), ultrasonography (two studies), and pQCT (four studies). Most of the skeletal muscle assessments were performed in the rectus femoris or lower leg muscles (four studies each). Several cross-sectional studies using CT imaging have linked lower muscle density in the hip and thigh to a history of falls [22,24]. An index of trunk muscle density was examined for its association with fall risk, but no significant correlation was identified [26]. An MRI-based study found that a higher percentage of adipose tissue per thigh area was linked to fall history [56]. Similarly, an analysis using the Goutallier classification determined that only gluteus medius infiltration was associated with fall history [57]. An ultrasonography study identified a combination of high rectus femoris echo intensity and low muscle mass as a significant predictor of future falls [71]. Studies using pQCT to assess lower leg muscle density have also reported associations with both fall history and future fall risk [109,110,112]. Regarding the risk of bias assessment, seven studies were rated as high quality, while four studies were rated as low quality (Figure 2). Of these, all prospective cohort studies were judged to be of low quality.

**Table 2:** Association between fatty infiltration of muscle and incidence of falls pQCT, peripheral quantitative CT

Author	Year	Study population, sample size (female, %), and age	Study design	Medical imaging modalities	Target muscle	Summary of the results
Inacio M [22]	2014	Community participants, sample size: 58 (female: 32, 55.2%), age: 74.0 ± 1.1 years	Cross‐sectional	CT	Psoas major, gluteus maximus, gluteus medius, gluteus minimus, vastus lateralis, rectus femoris, hamstrings, and hip adductor muscles	Fallers had significantly lower muscle density in the psoas major, gluteus maximus, gluteus medius and minimus, vastus lateralis, and hip adductor muscles than nonfallers (P < 0.05).
Anderson DE [23]	2016	Community participants, sample size: 174 (female: 117, 67.2%), age: 81.9 ± 6.4 years	Prospective	CT	Paraspinal muscles, posterior abdominal muscles, and anterior abdominal muscles	No significant association was observed between muscle density and falls in both male and female participants. For males, all trunk muscles (per 1 SD) (OR: 1.44, 95% CI: 0.69–2.97), paraspinal muscles (OR: 2.11, 95% CI: 0.91–4.89), posterior abdominal muscles (OR: 0.81, 95% CI: 0.41–1.60), anterior abdominal muscles (OR: 0.87, 95% CI: 0.43–1.80). For females: all trunk muscles (OR: 1.05, 95% CI: 0.74–1.48), paraspinal muscles (OR: 1.05, 95% CI: 0.77–1.44), posterior abdominal muscles (OR: 0.83, 95% CI: 0.61–1.12), anterior abdominal muscles (OR: 1.16, 95% CI: 0.84–1.60).
Erlandson KM [24]	2022	People infected with HIV, sample size: 569 (female: 183, 32.2%), age (mean): male, 60 years; female, 50 years	Cross‐sectional	CT	Rectus abdominis, anterolateral abdominal wall muscles, psoas major, paraspinal muscles, hamstrings, and quadriceps	In female participants, lower muscle density in the hamstrings was associated with increased odds of falling (OR: 0.94, 95% CI: 0.90–1.00). In male participants, no significant association was observed between muscle density and falls.
Linge J [56]	2020	Community participants, sample size: 9615 (female: 5046, 52.5%), age: 62.6 ± 7.5 years	Cross‐sectional	MRI	Thigh muscles	A higher proportion of adipose tissue in the thigh muscles was associated with an increased risk of falling (OR: 1.13, 95% CI: 1.07–1.20). In the sex-stratified analysis, such an association was observed in females (OR: 1.13, 95% CI: 1.07–1.20) but not in males (OR: 1.08, 95% CI: 0.96–1.19).
Kiyoshige Y [57]	2015	Hospitalized participants, sample size: 83 (female: 64, 77.1%), age: case, 83.3 (67–97) years; control, 75.0 (66–86) years	Case-control	MRI	Psoas major, gluteus maximus, gluteus medius, gluteus minimus, rectus femoris, hip adductor muscles, and obturator externus	The higher Goutallier stage of the gluteus medius was associated with increased odds of falling (OR: 3.2, 95% CI: 1.14–8.94).
Yamada M [71]	2022	Community participants, sample size: 773 (female: 414, 53.6%), age: 73.8 ± 6.0 years	Prospective	Ultrasonography	Rectus femoris and vastus intermedius	Older adults with lower muscle quality and quantity had significantly increased risks of falling according to multivariate analyses using older adults with better muscle quality and quantity as reference (adjusted HR: 1.54 (95% CI: 1.06–2.23)).
Sugita Y [72]	2018	Community participants, sample size: 152 (unclear), age: faller, 76.3 ± 4.5 years; nonfaller, 75.8 ± 5.6 years	Cross‐sectional	Ultrasonography	Rectus femoris and vastus intermedius	No significant association was observed between the echo intensity of the rectus femoris and vastus intermedius and falls (OR: 2.66, 95% CI: 0.61–16.12).
Frank-Wilson AW [109]	2016	Community participants, sample size: 169 (female: 126, 74.6%), age: 74 (60–98) years	Cross‐sectional	pQCT	Lower leg muscles	Lower muscle density in the lower leg was associated with increased odds of falling (OR: 0.81, 95% CI: 0.67–0.97).
Frank AW [110]	2015	Community participants, sample size: 113 (female: 113, 100%), age: 74.3 ± 7.7 years	Cross‐sectional	pQCT	Lower leg muscles	The muscle density in the lower leg of the fallers was lower than that of the non-fallers, with a decrease of 2.1 mg/cm³ (P = 0.02).
Laskou F [111]	2022	Community participants, sample size: 376 (female: 178, 47.5%), age: male, 63.9 ± 2.5 years; female, 65.6 ± 2.6 years	Cross‐sectional	pQCT	Lower leg muscles	No significant association was observed between lower leg muscle density and falls (OR per 1 SD decrease: 0.98, 95% CI: 0.75–1.27).
Scott D [112]	2019	Community participants, sample size: 2214 (female: 1550, 70.0%), age (mean): 70 years	Prospective	pQCT	Lower leg muscles	Lower muscle density in the lower leg was associated with an increased risk of multiple falls over a 12-month period (OR per 1 SD decrease: 1.44, 95% CI: 1.01–2.05). No significant association was observed between muscle density and single falls (six months (OR: 1.03; 95% CI: 0.87–1.22), 12 months (OR: 1.13, 95% CI: 1.00–1.29)).

Association between fatty infiltration of muscle and fall-related outcomes

Comfortable Walking Speed

**Figure 3:**
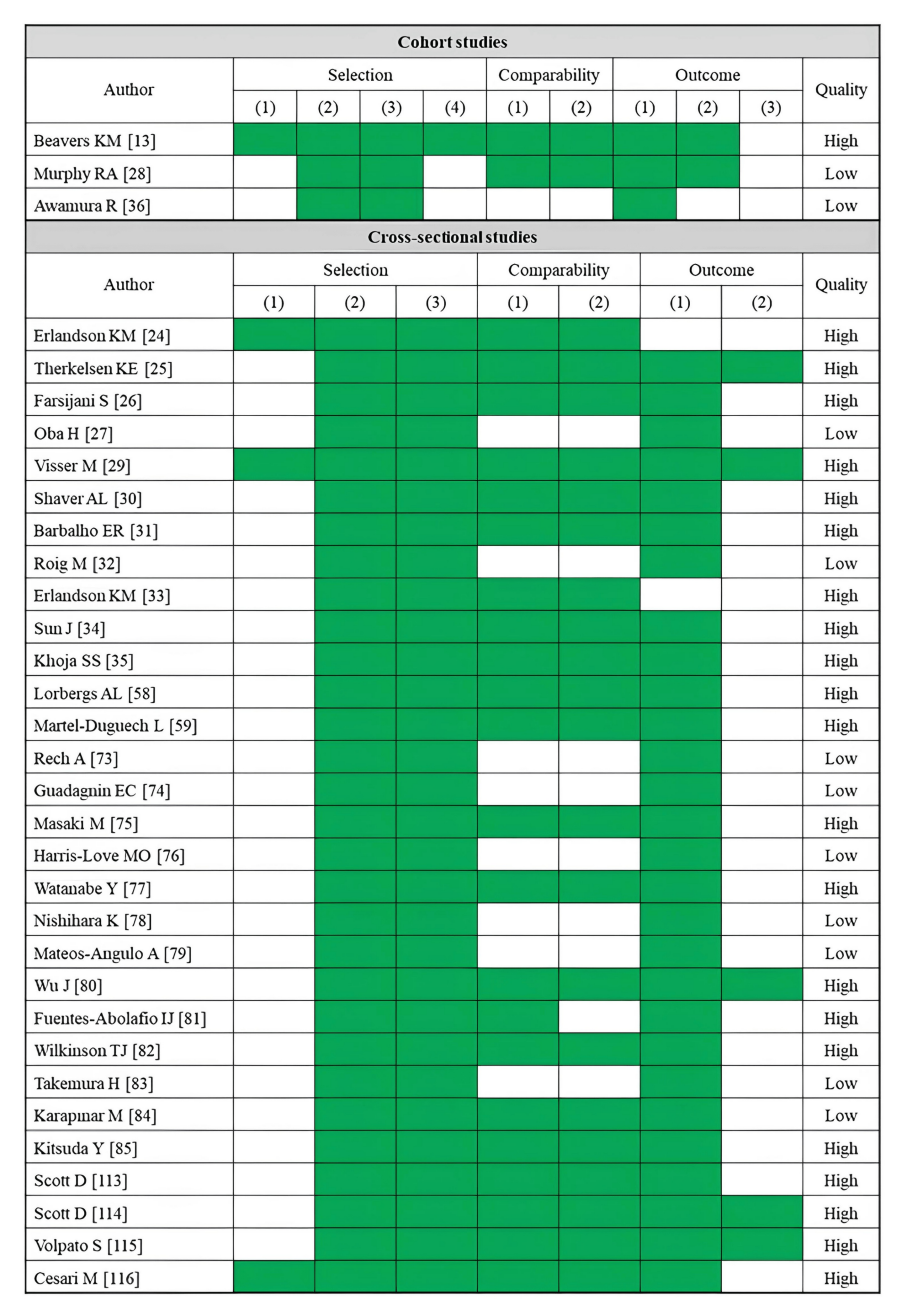
Assessment of risk of bias in studies on comfortable walking speed

A total of 33 studies reported comfortable walking speed as an outcome (Table 3). Fatty infiltration of muscle was assessed by CT (14 studies), MRI (two studies), ultrasonography (13 studies), and pQCT (four studies). The rectus femoris was the most assessed skeletal muscle (10 studies), followed by the whole thigh muscle (seven studies). Several CT-based cross-sectional and cohort studies have consistently reported that lower muscle density or a higher proportion of adipose tissue in the trunk, gluteal muscles, and thigh muscles is associated with reduced comfortable walking speed [13,25,26,28-31,35,36]. MRI-based studies linked a higher proportion of adipose tissue in the thigh muscles to a reduced comfortable walking speed [59], whereas no significant association existed in the lower leg muscles [58]. Most ultrasonography-based studies have used the rectus femoris muscle as an indicator. However, only a few studies have demonstrated a significant association between reduced comfortable walking speed and high echo intensity in the rectus femoris muscle [79,83,84]. Two pQCT-based studies reported a relationship between decreased muscle density in the lower legs and reduced comfortable walking speed [115,116]. Regarding the risk of bias assessment, 22 studies were rated as high quality, while 11 studies were rated as low quality (Figure 3).

**Table 3:** Association between fatty infiltration of muscle and comfortable walking speed β, standardized regression coefficient; COPD, chronic obstructive pulmonary disease; pQCT, peripheral quantitative CT

Author	Year	Study population, sample size (female, %), and age	Study design	Medical imaging modalities	Target muscle	Summary of the results
Beavers KM [13]	2013	Community participants, sample size: 2306 (female: 1158, 50.2%), age: 74.6 ± 2.9 years	Prospective	CT	Thigh muscles	Increased adipose tissue in the thigh muscles was associated with decreased walking speed (β ± SE: all participants, −0.009; males, −0.0119; females, −0.0168).
Erlandson KM [24]	2022	Persons infected with HIV, sample size: 571 (female: 184, 32.2%), age (range): 40–69 years	Cross‐sectional	CT	Rectus abdominis, anterolateral abdominal wall muscles, psoas major, paraspinal muscles, hamstrings, and quadriceps	In HIV-negative males, lower muscle density in the anterolateral abdominal wall muscles (β: 0.0034) and quadriceps (β: 0.0101) was associated with reduced comfortable walking speed. In HIV-positive males, the lower muscle density in the anterolateral abdominal wall muscles (β: 0.0026), psoas major (β: 0.0036), and hamstrings (β: 0.0048) was associated with reduced comfortable walking speed. In HIV-negative females, the lower muscle density in the anterolateral abdominal wall muscles (β: −0.0025) was associated with reduced comfortable walking speed, whereas in HIV-positive females, the lower muscle density in the rectus abdominis (β: 0.0011) was associated with reduced comfortable walking speed.
Therkelsen KE [25]	2016	Community participants, sample size: 1152 (female: 648, 56.2%), age: 66.2 ± 8.8 years	Cross‐sectional	CT	Trunk muscles	In the analysis of the participants, no significant association was observed between trunk muscle density and comfortable walking speed (OR: 1.21, 95% CI: 0.99–1.49). In the sex-stratified analysis, lower trunk muscle density was associated with reduced comfortable walking speed (walking speed ≤ 1 m/s) in both males (OR: 1.31, 95% CI: 1.03–1.67) and females (OR: 1.07, 95% CI: 1.01–1.45).
Farsijani S [26]	2021	Community participants, sample size: 1897 (female: 991, 52.2%), age: 78 (73–85) years	Cross‐sectional	CT	Thigh muscles	Greater adipose tissue area in the thigh muscles was associated with reduced comfortable walking speed (β: −0.018, 95% CI not reported).
Oba H [27]	2021	Community participants, sample size: 214 (female: 136, 63.7%), age (median): male, 78.3 years; female, 78.4 years	Cross‐sectional	CT	Quadriceps	Lower muscle density of the quadriceps was associated with reduced comfortable walking speed (r: 0.43).
Murphy RA [28]	2014	Community participants, sample size: 1542 (female: 830, 53.8%), age: 74.2 ± 2.9 years	Prospective	CT	Thigh muscles	In males, lower muscle density in the thigh was associated with reduced comfortable walking speed (walking speed ≤ 1 m/s) (OR: 0.8, 95% CI: 0.67–0.97), whereas no such association was observed in females (OR: 0.96, 95% CI: 0.81–1.14).
Visser M [29]	2002	Community participants, sample size: 2979 (female: 1537, 51.6%), age (range): 70–79 years	Cross‐sectional	CT	Thigh muscles	Lower muscle density in the thigh was associated with reduced comfortable walking speed in White men (β: 0.156), Black men (β: 0.114), White women (β: 0.101), and Black women (β: 0.008).
Shaver AL [30]	2022	Patients with cancer (head and neck), sample size: 90 (female: 19, 21.1%), age: 64.0 ± 10.7 years	Cross‐sectional	CT	Trunk muscles	In the analysis of the participants, those with myosteatosis exhibited a decrease in comfortable walking speed (β: 0.11, 95% CI: 0.007–0.22). In the sex-stratified analysis, no significant association was observed between myosteatosis and comfortable walking speed in males (β: 0.11, 95% CI: −0.001–0.22) and females (β: 0.14, 95% CI: −0.44–0.71).
Barbalho ER [31]	2019	Patients with cancer (gastric), sample size: 167 (female: 70, 42.0%), age: 69.2 ± 7.8 years	Cross‐sectional	CT	Trunk muscles	In the analysis of the participants, lower trunk muscle density was associated with reduced comfortable walking speed (β: 0.01, 95% CI: 0.003–0.01). In the sex-stratified analysis, lower muscle density was associated with reduced comfortable walking speed in males (β: 0.01, 95% CI: 0.001–0.015) but not in females (β: −0.00, 95% CI: −0.007–0.004).
Roig M [32]	2011	Patients with COPD, sample size: 21 (female: 10, 47.6%), age: 71.3 ± 8.1 years	Cross‐sectional	CT	Thigh muscles	No significant correlation was observed between the adipose tissue area in the thigh muscles and comfortable walking speed (r: −0.43).
Erlandson KM [33]	2023	People infected with HIV, sample size: 139 (unclear), age: 51.0 (40–71) years	Cross‐sectional	CT	Paraspinal muscle	No significant association was observed between the muscle density of the paraspinal muscles and comfortable walking speed (β: −0.022, 95% CI: −0.062–0.018).
Sun J [34]	2024	Persons infected with HIV, sample size: 798 (female: 0, 0%), age (range): 40–70 years	Cross‐sectional	CT	Rectus abdominis, anterolateral abdominal wall muscles, psoas major, paraspinal muscles, hamstrings, and quadriceps	Lower muscle density in the rectus abdominis (β: 0.0017, 95% CI: 0.00079–0.0026), anterolateral muscles (β: 0.0023, 95% CI: 0.0010–0.0036), psoas major (β: 0.0035, 95% CI: 0.0015–0.0055), paraspinal muscles (β: 0.0027, 95% CI: 0.0012–0.0042), hamstrings (β: 0.0037, 95% CI: 0.0019–0.0054), and quadriceps (β: 0.0023, 95% CI: 0.001–0.0036) was associated with reduced comfortable walking speed.
Khoja SS [35]	2018	Patients with rheumatoid arthritis, sample size: 60 (female: 49, 81.7%), age: 59.0 ± 9.8 years	Cross‐sectional	CT	Thigh muscles	Lower muscle density in the thigh was associated with reduced comfortable walking speed (β: −0.306, 95% CI: not reported).
Awamura R [36]	2020	Patients who underwent total hip arthroplasty, sample size: 18 (female: 18, 100%), age: unclear	Retrospective	CT	Gluteus maximus	A higher proportion of adipose tissue area in the gluteus maximus was associated with reduced comfortable walking speed (r: −0.71).
Lorbergs AL [58]	2015	Community participants, sample size: 35 (female: 35, 100%), age: 70 (60–75) years	Cross‐sectional	MRI	Tibialis anterior, soleus, and gastrocnemius	No significant association was observed between the adipose tissue area in the muscles and comfortable walking speed (tibialis anterior r: −0.33, soleus r: −0.07, gastrocnemius r: −0.17).
Martel-Duguech L [59]	2020	Patients with Cushing syndrome, sample size: 36 (female: 36, 100%), age: 51.0 ± 1.5 years	Cross‐sectional	MRI	Thigh muscles	A higher proportion of adipose tissue area in the thigh muscles was associated with reduced comfortable walking speed (β: −0.461, 95% CI: not reported).
Rech A [73]	2014	Community participants, sample size: 45 (female: 45, 100%), age: 70.3 ± 6.2 years	Cross‐sectional	Ultrasonography	Quadriceps	No significant correlation was observed between the echo intensity of the quadriceps and comfortable walking speed (r: −0.27).
Guadagnin EC [74]	2019	Community participants, sample size: 15 (female: 9, 60.0%), age: 75.4 ± 5.0 years	Cross‐sectional	Ultrasonography	Rectus femoris, vastus intermedius, biceps femoris, tibialis anterior, and medial gastrocnemius	No significant association was observed between the echo intensity of all lower limb muscles and comfortable walking speed (no statistical details reported).
Masaki M [75]	2016	Community participants, sample size: 35 (female: 35, 100%), age: 72.9 ± 7.4 years	Cross‐sectional	Ultrasonography	Erector spinae, lumbar multifidus, and psoas major	No significant association was observed between the echo intensity of all muscles and comfortable walking speed (no statistical details reported).
Harris-Love MO [76]	2018	Community participants, sample size: 30 (female: 0, 0%), age: 62.5 ± 9.2 years	Cross‐sectional	Ultrasonography	Rectus femoris	No significant association was observed between the echo intensity of the rectus femoris and comfortable walking speed (no detailed statistical description was provided).
Watanabe Y [77]	2019	Community participants, sample size: 53 (female: 28, 52.8%), age: male, 77.0 ± 5.5 years; female, 79.1 ± 6.1 years	Cross‐sectional	Ultrasonography	Quadriceps	Higher echo intensity of the quadriceps was associated with reduced comfortable walking speed (r: −0.362).
Nishihara K [78]	2016	Community participants, sample size: 19 (female: 5, 26.3%), age (range): 65–78 years	Cross‐sectional	Ultrasonography	Rectus femoris and vastus intermedius	No significant association was observed between the echo intensity of the rectus femoris (r: −0.26) and vastus intermedius (r: 0.16) and comfortable walking speed.
Mateos-Angulo A [79]	2021	Nursing home participants, sample size: 20 (female: 15, 75.0%), age: 85.4 ± 7.0 years	Cross‐sectional	Ultrasonography	Rectus femoris, vastus lateralis, medial gastrocnemius, and tibialis anterior	Higher lower limb echo intensity (summed value) was associated with reduced comfortable walking speed (β: −0.5, 95% CI: not reported).
Wu J [80]	2022	Individuals undergoing dialysis, sample size: 107 (female: 40, 37.4%), age: 53.5 ± 12.5 years	Cross‐sectional	Ultrasonography	Rectus femoris	No significant association was observed between the echo intensity of the rectus femoris and comfortable walking speed (β: −0.002, 95% CI: not reported).
Fuentes-Abolafio IJ [81]	2022	Patients with heart failure, sample size: 70 (female: 40, 57.1%), age: unclear	Cross‐sectional	Ultrasonography	Rectus femoris and vastus intermedius	No significant association was observed between the echo intensity of the rectus femoris and vastus intermedius and comfortable walking speed (males, r: −0.183; females, r: 0.18).
Wilkinson TJ [82]	2019	Patients with renal failure, sample size: 29 (female: 12, 41.4%), age: 57.5 ± 17.9 years	Cross‐sectional	Ultrasonography	Rectus femoris	No significant association was observed between the echo intensity of the rectus femoris and comfortable walking speed (β: −0.055, 95% CI: −0.018–0.015).
Takemura H [83]	2022	Patients with COPD, sample size: 17 (female: 2, 11.8%), age (mean): 83 years	Cross‐sectional	Ultrasonography	Rectus femoris	Higher echo intensity of the rectus femoris was associated with reduced comfortable walking speed (r: −0.65).
Karapınar M [84]	2024	Patients with knee osteoarthritis, sample size: 72 (female: 72, 100%), age: 51 (50–59) years	Cross‐sectional	Ultrasonography	Rectus femoris, vastus intermedius, vastus lateralis, vastus medialis, and hamstrings	Higher echo intensity of the rectus femoris (r: 0.204), vastus intermedius (r: 0.301), vastus medialis (r: 0.206), and biceps femoris (r: 0.445) was associated with slower comfortable walking time. No significant correlation was observed between echo intensity and comfortable walking time for the vastus lateralis (r: 0.092), semitendinosus (r: 0.004), or semimembranosus (r: 0.242).
Kitsuda Y [85]	2019	Patients who underwent total knee arthroplasty, sample size: 50 (female: 44, 88.0%), age: 76 (71–81) years	Cross‐sectional	Ultrasonography	Rectus femoris and vastus medialis	No significant association was observed between the echo intensity of the rectus femoris (β: −0.23, 95% CI: −0.87–0.42) or vastus medialis (β: 0.17, 95% CI: −0.33–0.66) and comfortable walking speed.
Scott D [113]	2018	Community participants, sample size: 85 (female: 49, 57.6%), age: 62.8 ± 7.9 years	Cross‐sectional	pQCT	Lower leg muscles	No significant association was observed between lower leg muscle density and comfortable walking speed (β: 0.003, 95% CI: −0.008–0.014).
Scott D [114]	2015	Community participants, sample size: 48 (female: 25, 52.1%), age: 71.6 ± 4.8 years	Cross‐sectional	pQCT	Lower leg muscles	No significant association was observed between lower leg muscle density and comfortable walking speed (β: 0.016, 95% CI: −0.009–0.04).
Volpato S [115]	2012	Community participants, sample size: 835 (female: 338, 40.4%), age (mean): 73.8 years	Cross‐sectional	pQCT	Lower leg muscles	The lower muscle density in the lower leg was associated with reduced comfortable walking speed (β: −0.102, 95% CI: not reported).
Cesari M [116]	2006	Community participants, sample size: 923 (female: 516, 55.9%), age: 74.8 ± 6.8 years	Cross‐sectional	pQCT	Lower leg muscles	The 3 SD increase in the lower leg muscle density was associated with a decreased likelihood of reduced comfortable walking speed (≤5th percentile) (OR: 0.67, 95% CI: 0.55–0.82).

Maximum Walking Speed

**Figure 4:**
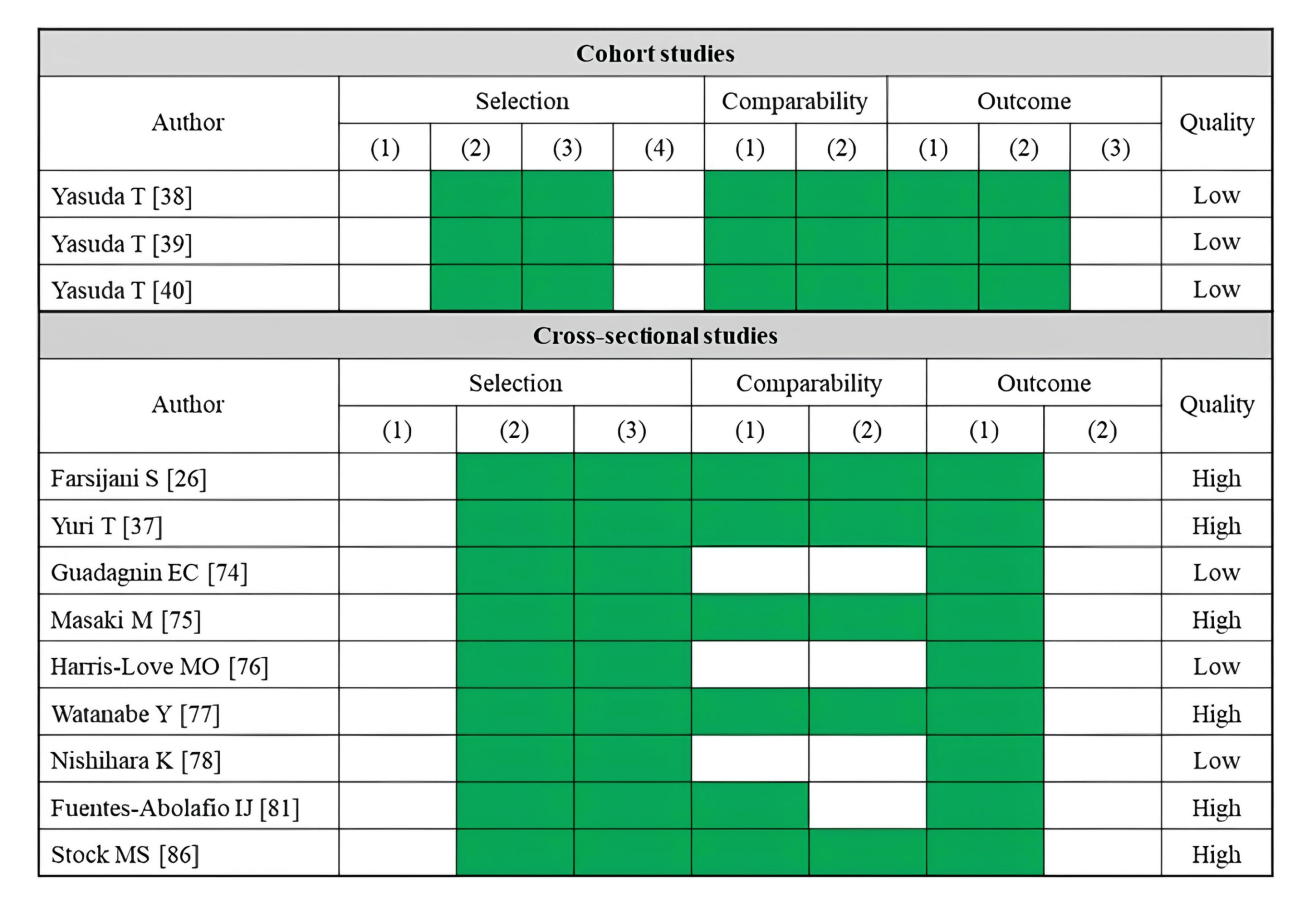
Assessment of risk of bias in studies on maximum walking speed

A total of 12 studies reported maximum walking speed as an outcome (Table 4). Fatty infiltration of muscle was assessed by CT (five studies) and ultrasonography (seven studies). The rectus femoris was the most assessed skeletal muscle (five studies), followed by the gluteus medius (four studies). Most CT-based studies have demonstrated significant associations between lower muscle density or a higher adipose tissue area in the thigh and gluteal muscle groups and reduced maximum walking speed [26,37,38]. However, the results varied between studies depending on the specific muscle area evaluated. An ultrasonography study reported a significant association between higher echo intensity in the quadriceps and reduced maximum walking speed [77]. No studies have reported significant associations between echo intensity and maximum walking speed in the muscles at other sites. Regarding the risk of bias assessment, six studies were rated as high quality, while six studies were rated as low quality (Figure 4).

**Table 4:** Association between fatty infiltration of muscle and maximum walking speed β, standardized regression coefficient

Author	Year	Study population, sample size (female, %), and age	Study design	Medical imaging modalities	Target muscle	Summary of the results
Farsijani S [26]	2021	Community participants, sample size: 1897 (female: 991, 52.2%), age: 78 (73–85) years	Cross‐sectional	CT	Thigh muscles	The higher proportion of adipose tissue area in the thigh muscles was associated with reduced maximum walking speed (β: −0.0044, 95% CI: not reported).
Yuri T [37]	2023	Patients with hip osteoarthritis, sample size: 91 (female: 91, 100%), age: 65.9 ± 10.6 years	Cross‐sectional	CT	Gluteus medius and gluteus minimus	The lower muscle density in the anterior portion of the gluteus minimus on the affected side was associated with reduced maximum walking speed (β: 0.345, 95% CI: not reported). No significant association was observed between the muscle density of the gluteus medius on the affected side (β: 0.038, 95% CI: not reported) or the unaffected side (β: −0.035, 95% CI: not reported) and the maximum walking speed.
Yasuda T [38]	2023	Patients who underwent total knee arthroplasty, sample size: 45 (female: 36, 80.0%), age: 75 (58–87) years	Retrospective	CT	Gluteus medius and gluteus minimus, quadriceps, and hamstrings	The higher proportion of adipose tissue area in the hamstrings (r: −0.34) was associated with reduced maximum walking speed. No significant association was observed between the adipose tissue area of the gluteus medius and minimus (r: −0.29) or the quadriceps (r: −0.29) and the maximum walking speed.
Yasuda T [39]	2022	Patients who underwent total hip arthroplasty, sample size: 42 (female: 33, 78.6%), age: 70.9 (46–87) years	Retrospective	CT	Gluteus maximus, gluteus medius, and gluteus minimus	Stepwise analysis revealed that the adipose tissue area of the gluteus maximus, gluteus medius, and gluteus minimus factors was not associated with the maximum walking speed.
Yasuda T [40]	2023	Patients who underwent total hip arthroplasty, sample size: 58 (female: 45, 77.6%), age: 70.9 ± 9.5 years	Retrospective	CT	Gluteus maximus, gluteus medius, and gluteus minimus	Stepwise analysis revealed that the adipose tissue area of the gluteus maximus, gluteus medius, and gluteus minimus factors was not associated with the maximum walking speed.
Guadagnin EC [74]	2019	Community participants, sample size: 15 (female: 9, 60%), age: 75.4 ± 5.0 years	Cross‐sectional	Ultrasonography	Rectus femoris, vastus intermedius, biceps femoris, tibialis anterior, and medial gastrocnemius	No significant association was observed between the echo intensity of the measured muscles and maximum walking time (no statistical details were reported due to stepwise analysis).
Masaki M [75]	2016	Community participants, sample size: 35 (female: 35, 100%), age: 72.9 ± 7.4 years	Cross‐sectional	Ultrasonography	Erector spinae, lumbar multifidus, and psoas major	No significant association was observed between the echo intensity of the measured muscles and the maximum walking speed (no statistical details reported).
Harris-Love MO [76]	2018	Community participants, sample size: 30 (female: 0, 0%), age: 62.5 ± 9.2 years	Cross‐sectional	Ultrasonography	Rectus femoris	No significant association was observed between the echo intensity of the rectus femoris and the maximum walking speed (no statistical details reported).
Watanabe Y [77]	2019	Community participants, sample size: 53 (female: 28, 52.8%), age: male, 77.0 ± 5.5 years; female, 79.1 ± 6.1 years	Cross‐sectional	Ultrasonography	Quadriceps	The echo intensity of the quadriceps was positively correlated with the maximum walking time (r: 0.29).
Nishihara K [78]	2016	Community participants, sample size: 19 (female: 5, 26.3%), age (range): 65–78 years	Cross‐sectional	Ultrasonography	Rectus femoris and vastus intermedius	No significant association was observed between the echo intensity of the rectus femoris and vastus intermedius and the maximum walking speed (rectus femoris, r: −0.18; vastus intermedius, r: −0.04).
Fuentes-Abolafio IJ [81]	2022	Patients with heart failure, sample size: 70 (female: 40, 57.1%), age: unclear	Cross‐sectional	Ultrasonography	Rectus femoris and vastus intermedius	No significant association was observed between the echo intensity of the rectus femoris and vastus intermedius and the maximum walking speed (males, r: 0.071; females, r: 0.175).
Stock MS [86]	2018	Community participants, sample size: 23 (female: 12, 52.2%), age: male, 74.0 ± 7.0 years; female, 71.0 ± 5.0 years	Cross‐sectional	Ultrasonography	Rectus femoris	No significant association was observed between the echo intensity of the rectus femoris and the maximum walking speed (unadjusted echo intensity, r: −0.046; adjusted echo intensity, r: −0.409).

TUG

**Figure 5:**
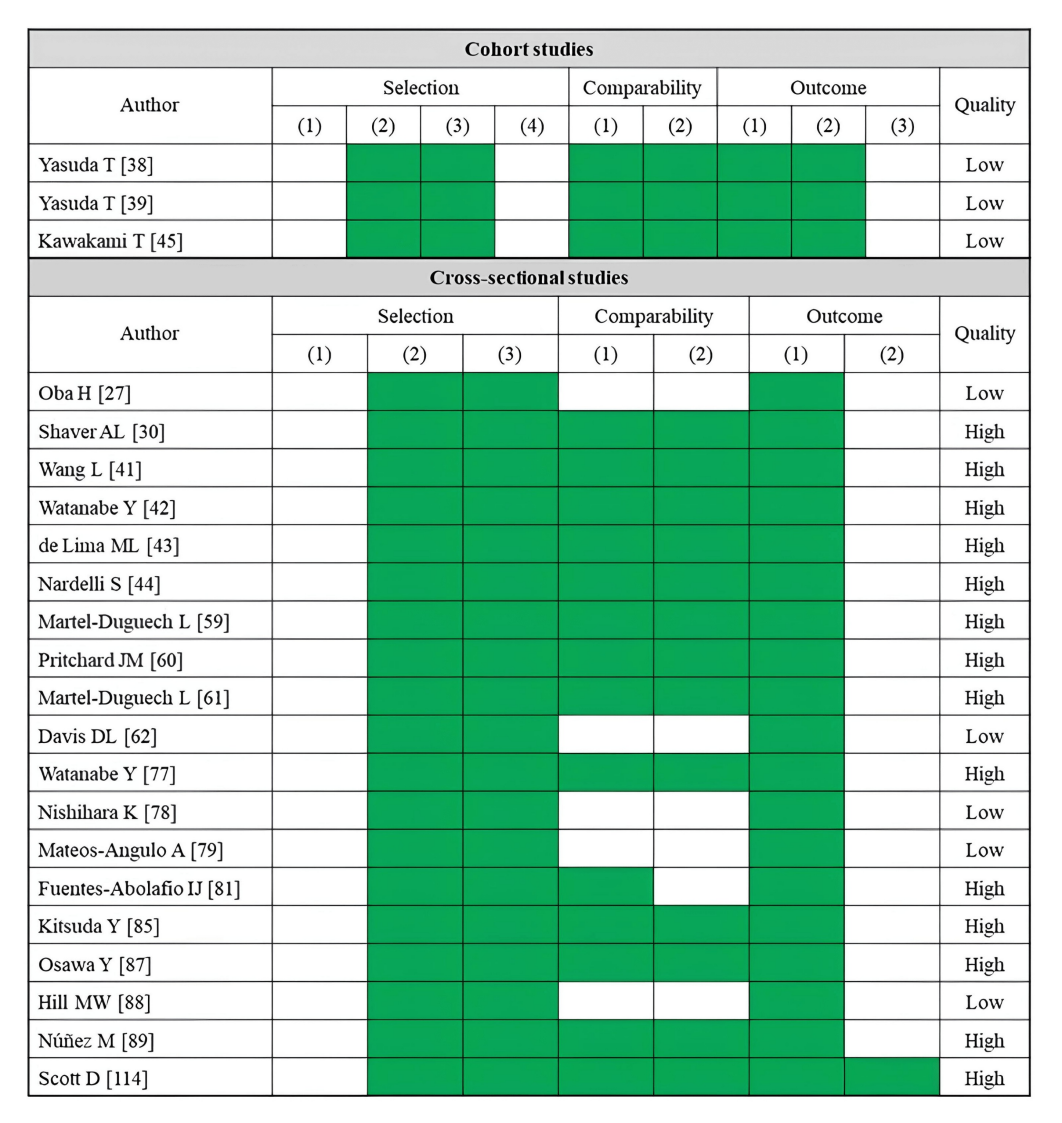
Assessment of risk of bias in studies on the TUG TUG, timed up and go test

A total of 22 studies reported TUG (Table 5). Fatty infiltration of muscle was assessed by CT (nine studies), MRI (four studies), ultrasonography (nine studies), and pQCT (one study). The rectus femoris was the most assessed skeletal muscle (seven studies), followed by the gluteus medius (five studies). Most CT-based studies uncovered significant associations between slower TUG time and lower muscle density or a higher adipose tissue area in the trunk, thigh, and gluteal muscle groups [30,38,41,43,44]. MRI-based studies linked a higher proportion of adipose tissue in the gluteus maximus and thigh muscles to slower TUG time [59,61,62], with no significant association existing in the lower leg muscles [60]. The relationship between echo intensity of the rectus femoris or quadriceps and TUG time was examined in seven studies, but only two reported a significant association [77,89]. A pQCT-based study found no significant association between lower leg muscle density and TUG time [114]. Regarding the risk of bias assessment, 14 studies were rated as high quality, while eight studies were rated as low quality (Figure 5).

**Table 5:** Association between fatty infiltration of muscle and the TUG β, standardized regression coefficient; pQCT, peripheral quantitative CT; TUG, timed up and go test

Author	Year	Study population, sample size (female, %), and age	Study design	Medical imaging modalities	Target muscle	Summary of the results
Oba H [27]	2021	Community participants, sample size: 214 (female: 136, 63.7%), age (median): male, 78.3 years; female, 78.4 years	Cross‐sectional	CT	Quadriceps	No significant correlation was observed between the muscle density of the quadriceps and TUG time (r: −0.34).
Shaver AL [30]	2022	Patients with cancer (head and neck), sample size: 90 (female: 19, 21.1%), age: 64.0 ± 10.7 years	Cross‐sectional	CT	Trunk muscles	In the analysis of the participants, individuals with myosteatosis exhibited slower TUG time (β: 1.16, 95% CI: 0.14–2.19). In sex-stratified analysis, myosteatosis was associated with slower TUG time in males (β: 1.34, 95% CI: 0.13–2.55) but not in females (β: 0.41, 95% CI: −1.62–2.45).
Yasuda T [38]	2023	Patients who underwent total knee arthroplasty, sample size: 45 (female: 36, 80.0%), age: 75.2 (58–87) years	Retrospective	CT	Gluteus medius and gluteus minimus, quadriceps, and hamstrings	The higher proportion of adipose tissue area in the hamstrings (r: 0.4) was associated with slower TUG time. No significant correlation was observed between the adipose tissue area of the gluteus medius and minimus (r: 0.18) or the quadriceps (r: 0.26) and TUG time.
Yasuda T [39]	2022	Patients who underwent total hip arthroplasty, sample size: 42 (female: 33, 78.6%), age: 70.9 (46–87) years	Retrospective	CT	Gluteus maximus, gluteus medius, and gluteus minimus	Stepwise analysis revealed that the adipose tissue area of the gluteus maximus, gluteus medius, and gluteus minimus factors was not associated with TUG time.
Wang L [41]	2021	Community participants, sample size: 301 (female: 194, 64.5%), age: 68.4 ± 6.1 years	Cross‐sectional	CT	Gluteus maximus, gluteus medius, gluteus minimus, paraspinal muscle, trunk and thigh muscles	In males, no significant association was observed between muscle density and TUG time. In females, the lower muscle density in the gluteus maximus (β: −0.06, 95% CI: −0.095 to −0.025) and trunk muscles (β: −0.068, 95% CI: −0.130 to −0.006) was associated with slower TUG time.
Watanabe Y [42]	2021	Community participants, sample size: 31 (female: 13, 41.9%), age (range): 69–83 years	Cross‐sectional	CT and ultrasonography	CT, thigh muscles; ultrasonography, rectus femoris, and vastus intermedius	No significant association was observed between thigh muscle density and TUG time (β: −0.07, 95% CI: not reported). Similarly, no significant association was observed between the echo intensity of the rectus femoris and vastus intermedius and TUG time (β: 0.279, 95% CI: not reported).
de Lima ML [43]	2024	Patients with cancer (breast), sample size: 56 (female: 56, 100%), age: 58.5 ± 8.3 years	Cross‐sectional	CT	Quadriceps	The lower muscle density in the quadriceps was associated with slower TUG time (β: −0.05, 95% CI: not reported).
Nardelli S [44]	2022	Patients with liver cirrhosis, sample size: 50 (female: 14, 28.0%), age: unclear	Cross‐sectional	CT	Trunk muscles	Myosteatosis was associated with slower TUG time (≥14 s) (OR: 0.85, 95% CI: 0.74–0.97).
Kawakami T [45]	2024	Patients who underwent THA, sample size: 124 (female: 124, 100%), age: TUG fast, 65.3 ± 10.5 years; TUG slow, 74.4 ± 7.8 years	Retrospective	CT	Psoas major, gluteus medius	Stepwise analysis revealed that the muscle density of the psoas major and gluteus medius was not associated with TUG time.
Martel-Duguech L [59]	2020	Patients with Cushing’s disease, sample size: 36 (female: 36, 100%), age: 51.0 ± 1.5 years	Cross‐sectional	MRI	Thigh muscles	The higher proportion of adipose tissue area in the thigh muscles was associated with slower TUG time (anterior (β: 0.504, 95% CI: not reported), posterior (β: 0.653, 95% CI: not reported), anterior + posterior (β: 0.629, 95% CI: not reported)).
Pritchard JM [60]	2015	Community participants, sample size: 57 (female: 57, 100%), age: unclear	Cross‐sectional	MRI	Lower leg muscles	No significant association was observed between the adipose tissue area in the lower leg muscles and TUG time (β: 0.048, 95% CI: −0.061–0.158).
Martel-Duguech L [61]	2021	Patients with acromegaly, sample size: 36 (female: 22, 61.1%), age: 54.0 ± 8.0 years	Cross‐sectional	MRI	Thigh muscles	The higher proportion of adipose tissue area in the thigh muscles was associated with slower TUG time (β: 0.737, 95% CI: not reported).
Davis DL [62]	2023	Older adults with urinary incontinence, sample size: 19 (female: 19, 100%), age: 76.3 ± 4.8 years	Cross‐sectional	MRI	Gluteus maximus, gluteus medius, gluteus minimus	The higher proportion of adipose tissue area in the gluteus maximus (r: 0.52) was associated with slower TUG time. No significant correlation was observed between the adipose tissue area of the gluteus medius and minimus (r: 0.37) and TUG time.
Watanabe Y [77]	2019	Community participants, sample size: 53 (female: 28, 52.8%), age: male, 77.0 ± 5.5 years; female, 79.1 ± 6.1 years	Cross‐sectional	Ultrasonography	Quadriceps	The higher echo intensity of the quadriceps was associated with slower TUG time (r: 0.321).
Nishihara K [78]	2016	Community participants, sample size: 19 (female: 5, 26.3%), age (range): 65–78 years	Cross‐sectional	Ultrasonography	Rectus femoris and vastus intermedius	No significant correlation was observed between the echo intensity of the rectus femoris (r: −0.19) and vastus intermedius (r: 0.06) and TUG time.
Mateos-Angulo A [79]	2021	Nursing home participants, sample size: 20 (female: 15, 75.0%), age: 85.4 ± 7.0 years	Cross‐sectional	Ultrasonography	Rectus femoris, vastus lateralis, medial gastrocnemius, and tibialis anterior	Stepwise analysis revealed that the higher echo intensity of the medial head of the gastrocnemius was associated with slower TUG time (β: 0.567, 95% CI: not reported).
Fuentes-Abolafio IJ [81]	2022	Patients with heart failure, sample size: 70 (female: 40, 57.1%), age: unclear	Cross‐sectional	Ultrasonography	Rectus femoris	No significant correlation was observed between echo intensity and TUG time in both males (r: 0.082) and females (r: −0.224).
Kitsuda Y [85]	2019	Patients who underwent total knee arthroplasty, sample size: 50 (female: 44, 88.0%), age: 76 (71–81) years	Cross‐sectional	Ultrasonography	Rectus femoris and vastus medialis	No significant association was observed between the echo intensity of the rectus femoris (β: 0.32, 95% CI: −0.31–0.95) and the vastus medialis (β: −0.14, 95% CI: −0.63–0.35) and TUG time.
Osawa Y [87]	2016	Community participants, sample size: 108 (female: 56, 51.9%), age (range): 88–92 years	Cross‐sectional	Ultrasonography	Rectus femoris	No significant association was observed between the echo intensity of the rectus femoris and TUG time (β: −0.04, 95% CI: not reported).
Hill MW [88]	2021	Community participants, sample size: 21 (female: 9, 42.9%), age: 69.9 ± 4.3 years	Cross‐sectional	Ultrasonography	Vastus lateralis and medial gastrocnemius	The higher echo intensities in the vastus lateralis (right lower limb: r, 0.491; left lower limb: r, 0.518) and gastrocnemius (right lower limb: r, 0.459; left lower limb: r, 0.516) regions were associated with slower TUG time
Núñez M [89]	2019	Patients with knee osteoarthritis, sample size: 61 (female: 45, 73.8%), age: 69.7 ± 7.2 years	Cross‐sectional	Ultrasonography	Rectus femoris	The higher echo intensity of the rectus femoris was associated with slower TUG time (β: 0.15, 95% CI: 0.3–0.26).
Scott D [114]	2015	Community participants, sample size: 48 (female: 25, 52.1%), age: 71.6 ± 4.8 years	Cross‐sectional	pQCT	Lower leg muscles	No significant association was observed between lower leg muscle density and TUG time (β: −0.224, 95% CI: −0.439–0.049).

SPPB

**Figure 6:**
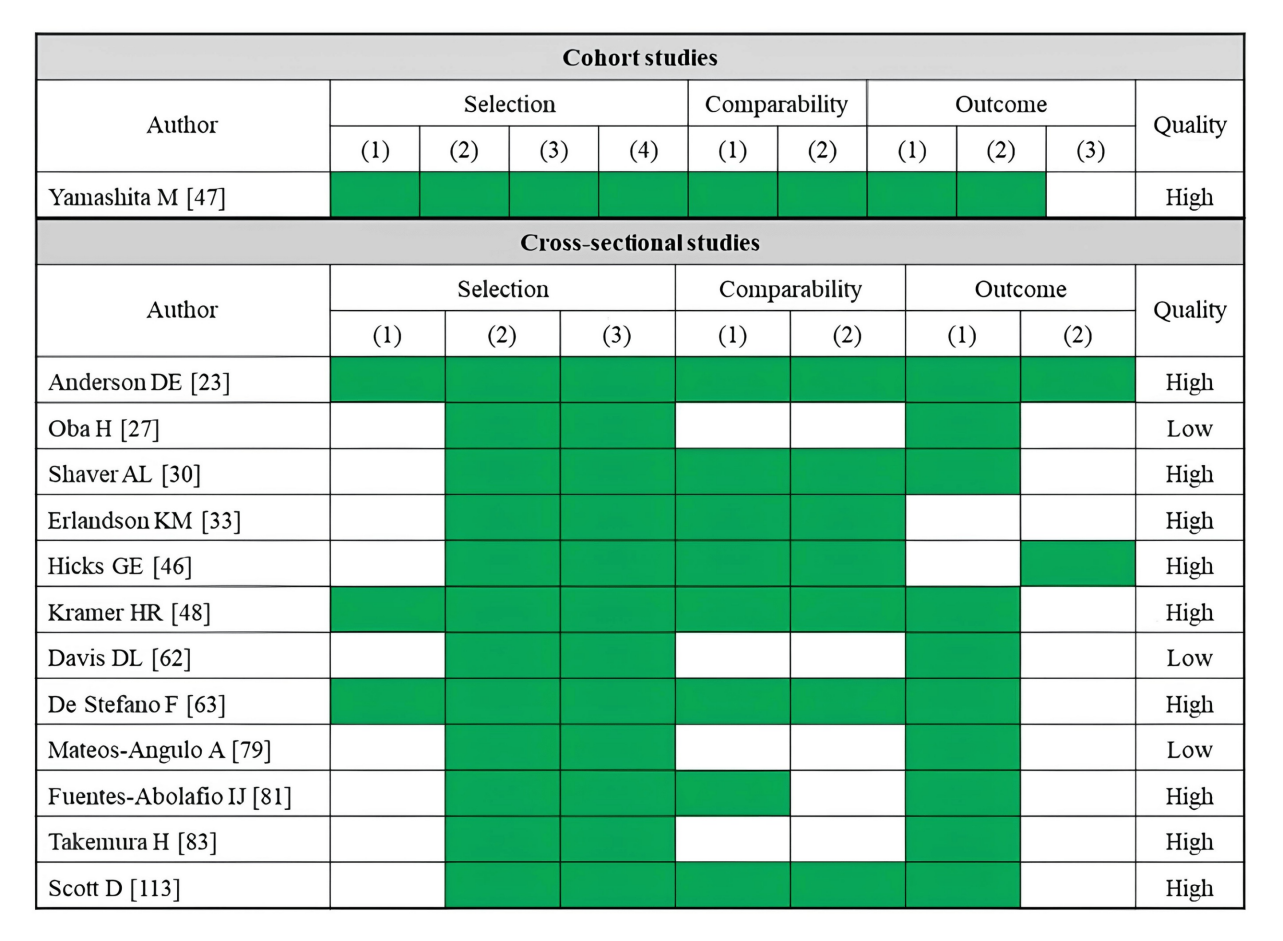
Assessment of risk of bias in studies on the SPBB SPBB, short physical performance battery

A total of 13 studies reported SPPB as an outcome (Table 6). Fatty infiltration of muscle was assessed by CT (seven studies), MRI (two studies), ultrasonography (three studies), and pQCT (one study). The paraspinal muscle and rectus femoris were the most assessed skeletal muscles, each examined in three studies. CT-based studies have consistently linked lower muscle density in the trunk and thigh muscles to a lower SPPB score [23,27,30,46,47,48]. MRI-based studies have suggested a correlation between a higher proportion of adipose tissue in the paraspinal muscles and a lower SPPB score [63], with no significant association existing in the gluteus muscle group [62]. Ultrasonography-based studies have linked higher echo intensity in the rectus femoris muscle to a lower SPPB score [79,83]. According to pQCT-based studies, the lower muscle density in the lower leg muscles and a lower SPPB score are related [113]. Regarding the risk of bias assessment, 10 studies were rated as high quality, while three studies were rated as low quality (Figure 6).

**Table 6:** Association between fatty infiltration of muscle and the SPPB β, standardized regression coefficient; COPD, chronic obstructive pulmonary disease; pQCT, peripheral quantitative CT; SPPB, short physical performance battery

Author	Year	Study population, sample size (female, %), and age	Study design	Medical imaging modalities	Target muscle	Summary of the results
Anderson DE [23]	2016	Community participants, sample size: 174 (female: 117, 67.2%), age: male, 83.7 ± 5.9 years; female, 81.1 ± 6.5 years	Cross‐sectional	CT	Paraspinal muscles, posterior abdominal muscles, and anterior abdominal muscles	In males, no significant association was observed between the measured muscle density and the SPPB score (paraspinal muscles (β: 0.48, 95% CI: −0.27–1.22), posterior abdominal muscle (β: 0.67, 95% CI: −0.09–1.44), anterior abdominal muscles (β: 0.15, 95% CI: −0.66–0.97), combined paraspinal and posterior and anterior abdominal muscles (β: 0.34, 95% CI: −0.45–1.14)). In females, the lower muscle density in the paraspinal muscles (β: 0.5, 95% CI: 0.01–0.98), posterior abdominal muscles (β: 1.03, 95% CI: 0.56–1.5), anterior abdominal muscles (β: 0.72, 95% CI: 0.24–1.19), and combined paraspinal and posterior and anterior abdominal muscles (β: 0.75, 95% CI: 0.24–1.26) was associated with the lower SPPB score.
Oba H [27]	2021	Community participants, sample size: 214 (female: 136, 63.7%), age (median): male, 78.3 years; female, 78.4 years	Cross‐sectional	CT	Quadriceps	The lower muscle density in the quadriceps was associated with a lower SPPB score (r: 0.35).
Shaver AL [30]	2022	Patients with cancer (head and neck), sample size: 90 (female: 19, 21.1%), age: 64 ± 10.7 years	Cross‐sectional	CT	Trunk muscles	In the analysis of all participants, myosteatosis was associated with the SPPB score (β: −1.49, 95% CI: −2.35 to −0.63). In the sex-stratified analysis, myosteatosis was associated with the SPPB score in males (β: −1.57, 95% CI: −2.46 to −0.68) but not in females (β: −0.86, 95% CI: −3.91 to 2.19).
Erlandson KM [33]	2023	People infected with HIV, sample size: 139 (unclear), age (range): 40–71 years	Cross‐sectional	CT	Paraspinal muscle	No significant association was observed between the paraspinal muscle density and the SPPB score (β: 0.011, 95% CI: −0.031–0.053).
Hicks GE [46]	2005	Community participants, sample size: 1527 (female: 788, 51.6%), age: male, 73.9 ± 2.9 years; female, 73.5 ± 2.9 years	Cross‐sectional	CT	Trunk and thigh muscles	The lower muscle density in both the trunk (β: 0.004, SE: 0.002) and the thigh (β: 0.0016, SE: 0.003) was associated with a lower SPPB score.
Yamashita M [47]	2022	Patients with aortic disease, sample size: 123 (female: 37, 30.1%), age: 70 (IQR, 58,77) years	Prospective	CT	Trunk muscles	A significant association was observed between the change in the trunk muscle density and the change in the SPPB score (β: 0.296, 95% CI: 0.066–0.4).
Kramer HR [48]	2012	Patients with rheumatoid arthritis, sample size: 152 (female: 98, 64.5%), age: 63.0 ± 8.0 years	Cross‐sectional	CT	Thigh muscles	The lower muscle density in the thigh was associated with the lower SPPB score (β: 0.36, 95% CI: not reported).
Davis DL [62]	2023	Older adults with urinary incontinence, sample size: 19 (female: 19, 100%), age: 76.3 ± 4.8 years	Cross‐sectional	MRI	Gluteus maximus, gluteus medius, gluteus minimus	No significant correlation was observed between the adipose tissue area of the gluteus medius and minimus (r: 0.29) and the gluteus maximus (r: −0.21) and the SPPB score.
De Stefano F [63]	2015	Community participants, sample size: 348 (unclear), age (range): unclear	Cross‐sectional	MRI	Paraspinal muscle	The higher proportion of adipose tissue area in the paraspinal muscle was associated with the lower SPPB score (β: −0.071, 95% CI: −0.042 to −0.017).
Mateos-Angulo A [79]	2021	Nursing home participants, sample size: 20 (female: 15, 75.0%), age: 85.4 ± 7 years	Cross‐sectional	Ultrasonography	Rectus femoris, vastus lateralis, medial gastrocnemius, and tibialis anterior	The higher echo intensity of the rectus femoris was associated with the lower SPPB score (β: 0.431, 95% CI: not reported).
Fuentes-Abolafio IJ [81]	2022	Patients with heart failure, sample size: 70 (female: 40, 57.1%), age: unclear	Cross‐sectional	Ultrasonography	Rectus femoris	No significant correlation was observed between the echo intensity of the rectus femoris and the SPPB score in both males (r: −0.222) and females (r: 0.224).
Takemura H [83]	2022	Patients with COPD, sample size: 17 (female: 2, 11.8%), age (mean): 83 years	Cross‐sectional	Ultrasonography	Rectus femoris	The higher echo intensity of the rectus femoris was associated with the lower SPPB score (r: −0.59).
Scott D [113]	2018	Community participants, sample size: 85 (female: 49, 57.6%), age: 62.8 ± 7.9 years	Cross‐sectional	pQCT	Lower leg muscles	The lower muscle density in the lower leg was associated with the lower SPPB score (β: 0.118, 95% CI: 0.007–0.228).

Muscle Strength

**Figure 7:**
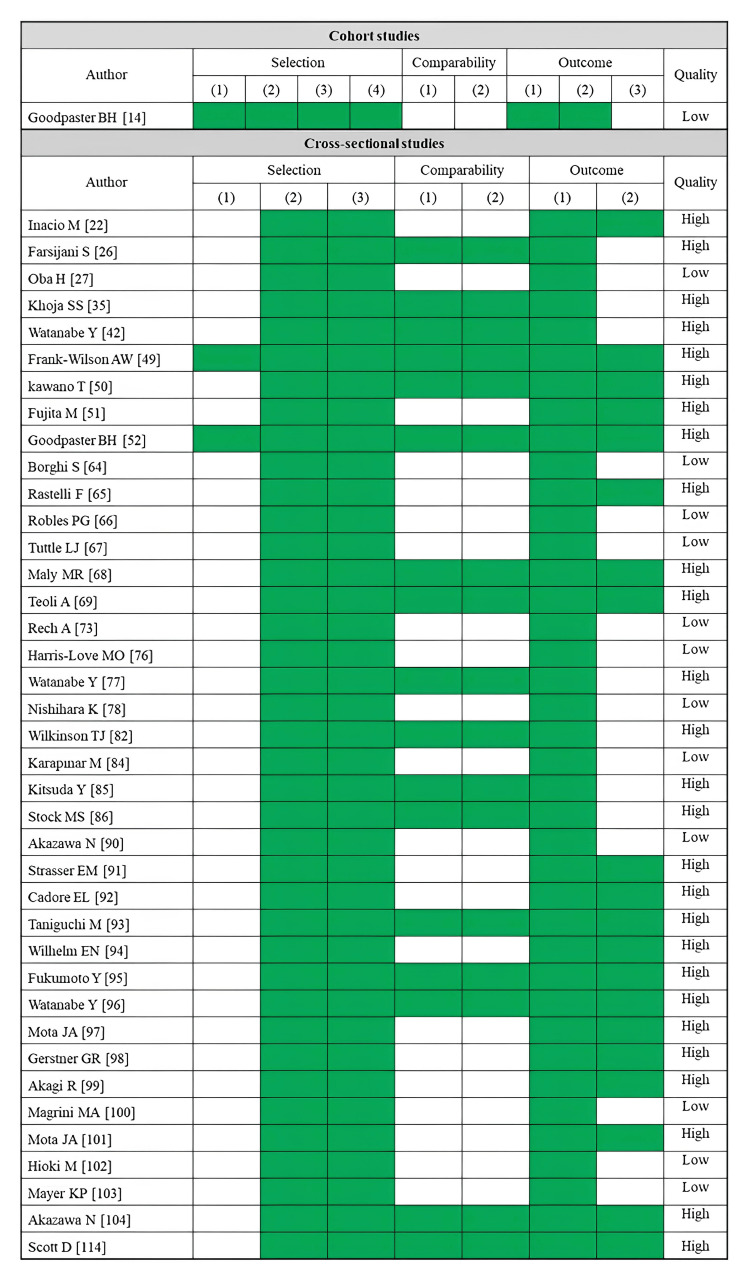
Assessment of risk of bias in studies on muscle strength

A total of 40 studies reported muscle strength as an outcome (Table 7). Fatty infiltration of muscle was assessed by CT (10 studies), MRI (six studies), ultrasonography (24 studies), and pQCT (one study). The rectus femoris was the most assessed skeletal muscle (16 studies), followed by the quadriceps (eight studies). In addition, most studies have used knee joint extensor strength as an outcome. Many cross-sectional studies using CT and MRI have linked lower muscle density and greater adipose tissue in the thigh and quadriceps to lower knee extension strength [27,35,49,52,65,66,68,69]. Nevertheless, the only longitudinal study found no significant association between thigh muscle density and changes in knee joint extensor strength [14]. Many ultrasonography-based studies have uncovered a correlation between higher echo intensity in the rectus femoris or quadriceps and lower knee extension strength [73,76,77,84,86,90,92-97,104]. Regarding the risk of bias assessment, 27 studies were rated as high quality, while 13 studies were rated as low quality (Figure 7).

**Table 7:** Association between fatty infiltration of muscle and muscle strength β, standardized regression coefficient; COPD, chronic obstructive pulmonary disease; pQCT, peripheral quantitative CT

Author	Year	Study population, sample size (female, %), and age	Study design	Medical imaging modalities	Target muscle	Summary of the results
Goodpaster BH [14]	2006	Community participants, sample size: 1880 (female: 951, 50.6%), age (range): 70–79 years	Prospective	CT	Thigh muscles	No significant association was found between thigh muscle density and changes in muscle strength in either male or female participants.
Inacio M [22]	2014	Community participants, sample size: 58 (female: 32, 55.2%), age: 74.4 ± 1.1 years	Cross‐sectional	CT	Psoas major, gluteus maximus, gluteus medius, gluteus minimus, vastus lateralis, rectus femoris, hamstrings, and hip adductor muscles	In the analysis of muscle density, significant positive correlations were observed between the gluteus medius + minimus and hip abduction strength (r: 0.53), the gluteus maximus and hip extension strength (r: 0.31), the psoas major and hip flexion strength (r: 0.34), and the hamstrings and knee flexion strength (r: 0.41). In the analysis of adipose tissue ratio, significant negative correlations were observed between the gluteus medius + minimus and hip abduction strength (r: −0.49), the gluteus maximus and hip extension strength (r: −0.39), and the psoas major and hip flexion strength (r: −0.46).
Farsijani S [26]	2021	Community participants, sample size: 1897 (female: 991, 52.2%), age: 78 (73–85) years	Cross‐sectional	CT	Thigh muscles	No significant association was observed between thigh muscle density and knee extension strength (β: −0.1201, 95% CI: not reported).
Oba H [27]	2021	Community participants, sample size: 214 (female: 136, 63.6%), age (median): male, 78.3 years; female, 78.4 years	Cross‐sectional	CT	Quadriceps	The lower muscle density in the quadriceps was associated with lower knee extension strength (r: 0.26).
Khoja SS [35]	2018	Patients with rheumatoid arthritis, sample size: 60 (female: 49, 81.7%), age: 59.0 ± 9.8 years	Cross‐sectional	CT	Thigh muscles	The lower muscle density in the thigh was associated with lower knee extension strength (β: 0.212, 95% CI: not reported).
Watanabe Y [42]	2021	Community participants, sample size: 31 (female: 13, 41.9%), age (range): 69–83 years	Cross‐sectional	CT and ultrasonography	CT, thigh muscles; ultrasonography, rectus femoris, and vastus intermedius	No significant correlation was observed between the muscle density of the thigh and knee extension strength (β: −0.175, 95% CI: not reported). Similarly, no significant correlation was observed between the echo intensity of the rectus femoris and knee extension strength (β: −0.321, 95% CI: not reported).
Frank-Wilson AW [49]	2018	Community participants, sample size: 4842 (female: 2801, 57.8%), age: male, 76 (72–81) years; female, 76 (72–80) years	Cross‐sectional	CT	Thigh muscles	The lower muscle density in the thigh was associated with lower knee extension strength (males (β: 0.22, 95% CI: 0.18–0.26; females (β: 0.2, 95% CI: 0.17–0.24)) and lower rate of force development in knee extension (males (β: 0.1, 95% CI: 0.05–0.14); females (β: 0.1, 95% CI: 0.07–0.15)). The higher proportion of adipose tissue area in the thigh muscles was associated with lower knee extension strength (males (β: −0.23, 95% CI: −0.27 to −0.19); females (β: −0.15, 95% CI: −0.18 to −0.12)) and lower rate of force development in knee extension (males (β: −0.09, 95% CI: −0.13 to −0.04); females (β: −0.11, 95% CI: −0.14 to −0.07)).
Kawano T [50]	2021	Patients with hip osteoarthritis, sample size: 108 (female: 108, 100%), age: 65.4 ± 10.4 years	Cross‐sectional	CT	Gluteus maximus, gluteus medius, and gluteus minimus	On the affected side, no significant association was observed between muscle density and hip abduction strength for the gluteus maximus (β: −0.128, 95% CI: −0.339–0.115), gluteus medius (β: 0.197, 95% CI: −0.097–0.487), or gluteus minimus (β: −0.032, 95% CI: −0.189–0.135). On the unaffected side, the lower muscle density in the gluteus medius (β: 0.44, 95% CI: 0.211–1.081) and gluteus minimus (β: 0.238, 95% CI: 0.055–0.505) was associated with lower hip abduction strength. No significant association was observed between the muscle density of the gluteus maximus and hip abduction strength (β: −0.196, 95% CI: −0.507–0.066).
Fujita M [51]	2017	Patients with hip fracture, sample size: 23 (female: 17, 73.9%), age: 84 (72–94) years	Cross‐sectional	CT	Gluteus medius	No significant association was observed between the adipose tissue area of the gluteus medius and hip abduction strength (no statistical details provided).
Goodpaster BH [52]	2001	Community participants, sample size: 2627 (female: 1342, 51.1%), age: 73.6 ± 2.9 years	Cross‐sectional	CT	Thigh muscles	The lower muscle density in the thigh was associated with lower knee extension strength in both males and females (β and 95% CI: not reported).
Borghi S [64]	2022	Community participants, sample size: 34 (female: 24, 70.6%), age: 65.6 ± 4.7 years	Cross‐sectional	MRI	Thigh muscles	The higher proportion of adipose tissue area in the thigh muscles was associated with lower knee extension strength (N) (r: −0.35). No significant correlation was observed between the adipose tissue area in the thigh muscles and knee extension strength in terms of 1RM.
Rastelli F [65]	2015	Community participants, sample size: 11 (female: 11, 100%), age: with obesity, 72.4 ± 2.3 years; without obesity, 72.7 ± 1.9 years	Cross‐sectional	MRI	Quadriceps	The higher proportion of adipose tissue area in the quadriceps was associated with lower knee extension strength per unit of quadriceps area (r: −0.825).
Robles PG [66]	2015	Patients with COPD, sample size: 10 (female: 5, 50.0%), age: 70.4 ± 6.7 years	Cross‐sectional	MRI	Vastus lateralis and soleus	The higher lipid/total proton ratio in the vastus lateralis was associated with lower isokinetic knee extension strength (r: −0.832) and lower isometric knee extension strength (r: −0.725). Similarly, the higher lipid/total proton ratio in the soleus muscle was associated with lower isokinetic knee extension strength (r: −0.813) and lower isometric knee extension strength (r: −0.798).
Tuttle LJ [67]	2011	Patients with diabetes mellitus, sample size: 22 (female: 7, 31.8%), age: 64.5 ± 12.7 years	Cross‐sectional	MRI	Lower leg muscles	The greater the volume of adipose tissue in the lower leg muscles, the lower the strength of the plantar flexion muscles of the ankle joint (r: −0.45), but there was no significant correlation with the rate of force development for plantar flexion of the ankle joint (r: −0.29). No significant correlation between the volume of adipose tissue in the lower leg muscles and the strength of the dorsiflexion muscles of the ankle joint (r: −0.06) or the rate of force development for dorsiflexion of the ankle joint (r: −0.02).
Maly MR [68]	2013	Patients with knee osteoarthritis, sample size: 73 (female: 73, 100%), age: 64.6 ± 6.7 years	Cross‐sectional	MRI	Thigh muscles	The greater adipose tissue volume in the thigh muscles was associated with lower knee extension strength (β: −0.225, 95% CI: not reported).
Teoli A [69]	2022	Patients with knee osteoarthritis, sample size: 41 (female: 24, 58.5%), age: unclear	Cross‐sectional	MRI	Vastus medialis	The higher proportion of adipose tissue area in the vastus medialis was associated with lower knee extension strength (B: −0.04, 95% CI: −0.072 to −0.007).
Rech A [73]	2014	Community participants, sample size: 45 (female: 45, 100%), age: 70.28 ± 6.2 years	Cross‐sectional	Ultrasonography	Quadriceps	Higher echo intensity of the quadriceps was associated with lower knee extension strength (r: −0.409) and lower rate of force development in knee extension (r: −0.386).
Harris-Love MO [76]	2018	Community participants, sample size: 30 (female: 0, 0%), age: 62.5 ± 9.2 years	Cross‐sectional	Ultrasonography	Rectus femoris	Higher echo intensity of the rectus femoris was associated with lower rate of force development in knee extension at 60°/s (r: −0.47) and 180°/s (r: −0.49).
Watanabe Y [77]	2019	Community participants, sample size: 53 (female: 28, 52.8%), age: male, 77.0 ± 5.5 years; female, 79.1 ± 6.1 years	Cross‐sectional	Ultrasonography	Quadriceps	Higher echo intensity of the quadriceps was associated with lower knee extension strength (r: −0.511).
Nishihara K [78]	2016	Community participants, sample size: 19 (female: 5, 26.3%), age (range): 65–78 years	Cross‐sectional	Ultrasonography	Rectus femoris and vastus intermedius	No significant correlation was observed between the echo intensity of the rectus femoris (r: −0.08) and vastus intermedius (r: −0.12) and knee extension strength.
Wilkinson TJ [82]	2019	Patients with renal failure, sample size: 29 (female: 12, 41.4%), age: 57.5 ± 17.9 years	Cross‐sectional	Ultrasonography	Rectus femoris	No significant association was observed between the echo intensity of the rectus femoris and knee extension strength (β: 0.137, 95% CI: −0.702–1.291).
Karapınar M [84]	2024	Patients with knee osteoarthritis, sample size: 72 (female: 72, 100%), age (range): 50–59 years	Cross‐sectional	Ultrasonography	Rectus femoris, vastus intermedius, vastus lateralis, vastus medialis, and hamstrings	Higher echo intensity of the rectus femoris (r: −0.164) and vastus medialis (r: −0.248) was associated with lower knee extension strength. No significant correlation was observed between the echo intensity of the vastus intermedius (r: −0.135), vastus lateralis (r: −0.033), biceps femoris (r: 0.141), semitendinosus (r: 0.138), and semimembranosus (r: 0.158) and knee extension strength. Higher echo intensity of the biceps femoris (r: 0.091) was associated with lower knee flexion strength. No significant correlation was observed between the echo intensity of the rectus femoris (r: −0.335), vastus intermedius (r: −0.092), vastus lateralis (r: −0.068), vastus medialis (r: −0.044), semitendinosus (r: 0.057), and semimembranosus (r: 0.106) and knee flexion strength.
Kitsuda Y [85]	2019	Patients who underwent total knee arthroplasty, sample size: 50 (female: 44, 88.0%), age: 76 (71–81) years	Cross‐sectional	Ultrasonography	Rectus femoris and vastus medialis	No significant association was observed between the echo intensity of the vastus medialis (β: −0.37, 95% CI: −1.10–0.36) or the rectus femoris (β: −0.04, 95% CI: −0.61–0.53) and knee extension strength.
Stock MS [86]	2018	Community participants, sample size: 23 (female: 12, 52.2%), age: male, 74.0 ± 7.0 years; female, 71.0 ± 5.0 years	Cross‐sectional	Ultrasonography	Rectus femoris	Higher adjusted echo intensity of the rectus femoris was associated with lower knee extension strength (r: −0.500), whereas no significant correlation was observed between unadjusted echo intensity and knee extension strength (r: −0.120).
Akazawa N [90]	2017	Community participants, sample size: 25 (female: 25, 100%), age: 83.4 ± 7.8 years	Cross‐sectional	Ultrasonography	Quadriceps	Higher echo intensity of the quadriceps was associated with lower knee extension strength (r: −0.635, 95% CI: not reported).
Strasser EM [91]	2013	Community participants, sample size: 25 (unclear), age: unclear	Cross‐sectional	Ultrasonography	Rectus femoris, vastus intermedius, vastus lateralis, and vastus medialis	The echo intensities of the rectus femoris (r: −0.301), vastus intermedius (r: −0.339), vastus lateralis (r: −0.272), and vastus medialis (r: −0.10) showed no significant correlations with knee extensor strength.
Cadore EL [92]	2012	Community participants, sample size: 31 (female: 25, 52.1%), age: 65.5 ± 5.0 years	Cross‐sectional	Ultrasonography	Quadriceps	Higher echo intensity of the quadriceps was associated with lower isometric knee extension strength (r: −0.51) and lower rate force development in knee extension at 60°/s (r: −0.48), 180°/s (r: −0.64), and 360°/s (r: −0.67).
Taniguchi M [93]	2017	Community participants, sample size: 179 (female: 179, 100%), age: 74.1 ± 4.9 years	Cross‐sectional	Ultrasonography	Rectus femoris	Higher echo intensity of the rectus femoris was associated with lower knee extension strength (β: −0.16, 95% CI: −0.702 to −0.01).
Wilhelm EN [94]	2014	Community participants, sample size: 50 (female: 0, 0%), age: 66.1 ± 4.5 years	Cross‐sectional	Ultrasonography	Quadriceps, rectus femoris, vastus intermedius, vastus lateralis, and vastus medialis	Higher echo intensity of the vastus lateralis (r: −0.551), rectus femoris (r: −0.46), vastus intermedius (r: −0.484), vastus medialis (r: −0.64), and overall quadriceps (r: −0.628) was associated with lower knee extension strength. Higher echo intensities of the vastus lateralis (r: −0.562), rectus femoris (r: −0.498), vastus intermedius (r: −0.501), vastus medialis (r: −0.656), and quadriceps (r: −0.657) were associated with lower 1RM knee extension strength.
Fukumoto Y [95]	2012	Community participants, sample size: 92 (female: 92, 100%), age: 70.4 ± 6.6 years	Cross‐sectional	Ultrasonography	Rectus femoris	Higher echo intensity of the rectus femoris was associated with lower knee extension strength (β: −0.27, 95% CI: −1.24 to −0.23).
Watanabe Y [96]	2013	Community participants, sample size: 184 (female: 0, 0%), age: 74.4 ± 5.9 years	Cross‐sectional	Ultrasonography	Rectus femoris	Higher echo intensity of the rectus femoris was associated with lower knee extension strength (β: −0.294, 95% CI: not reported).
Mota JA [97]	2017	Community participants, sample size: 13 (female: 0, 0%), age: 74.0 ± 6.0 years	Cross‐sectional	Ultrasonography	Rectus femoris	Higher echo intensity of the rectus femoris was associated with lower knee extension strength (r: −0.58).
Gerstner GR [98]	2017	Community participants, sample size: 20 (female: 0, 0%), age: 69.5 ± 3.1 years	Cross‐sectional	Ultrasonography	Medial and lateral gastrocnemius	Higher echo intensity of the gastrocnemius was associated with lower ankle plantarflexion strength (r: −0.526) and lower rate of force development in ankle plantarflexion (r: −0.524).
Akagi R [99]	2018	Community participants, sample size: 33 (female: 14, 42.4%), age: male, 73.0 ± 5.0 years; female, 72.0 ± 7.0 years	Cross‐sectional	Ultrasonography	Lateral gastrocnemius and soleus	Higher echo intensity of the lateral gastrocnemius was associated with lower ankle plantarflexion strength (β: −0.203, 95% CI: not reported).
Magrini MA [100]	2018	Community participants, sample size: 18 (female: 18, 100%), age: 74.9 ± 5.8 years	Cross‐sectional	Ultrasonography	Rectus femoris	No significant correlation was observed between the echo intensity of the rectus femoris and knee extension strength (r: −0.342).
Mota JA [101]	2018	Community participants, sample size: 22 (female: 0, 0%), age: 69.0 ± 3.0 years	Cross‐sectional	Ultrasonography	Medial and lateral gastrocnemius	Higher echo intensity of the gastrocnemius was associated with lower rate of force development in ankle plantarflexion (r: −0.491).
Hioki M [102]	2012	Participants in exercise classes, sample size: 8 (female: 18,100%), age (range): 60–79 years	Cross‐sectional	Ultrasonography	Rectus femoris	No significant correlation was observed between the echo intensity of the rectus femoris and knee extension strength (no statistical details provided).
Mayer KP [103]	2021	Patients with COPD, sample size: 12 (female: 4, 33.3%), age: 58 (IQR, 45.5, 65) years	Cross‐sectional	Ultrasonography	Rectus femoris and tibialis anterior	No significant correlation was observed between the echo intensity of the rectus femoris (r: −0.385) and tibialis anterior (r: 0.004) and the rate of force development during knee extension.
Akazawa N [104]	2018	Patients with stroke, sample size: 50 (female: 26, 52.0%), age: 78.7 ± 9.0 years	Cross‐sectional	Ultrasonography	Quadriceps	Higher echo intensity of the quadriceps on the paretic side was associated with lower knee extensor strength on the paretic side (β: −0.42, 95% CI: not reported). Similarly, higher echo intensity of the quadriceps on the nonparetic side was associated with lower knee extensor strength on the nonparetic side (β: −0.5, 95% CI: not reported).
Scott D [114]	2015	Community participants, sample size: 48 (female: 25, 52.1%), age: 71.6 ± 4.8 years	Cross‐sectional	pQCT	Lower leg muscles	No significant association was observed between the muscle density of the lower leg and knee extensor strength (β: 0.254, 95% CI: −0.146–0.655).

6MWT

**Figure 8:**
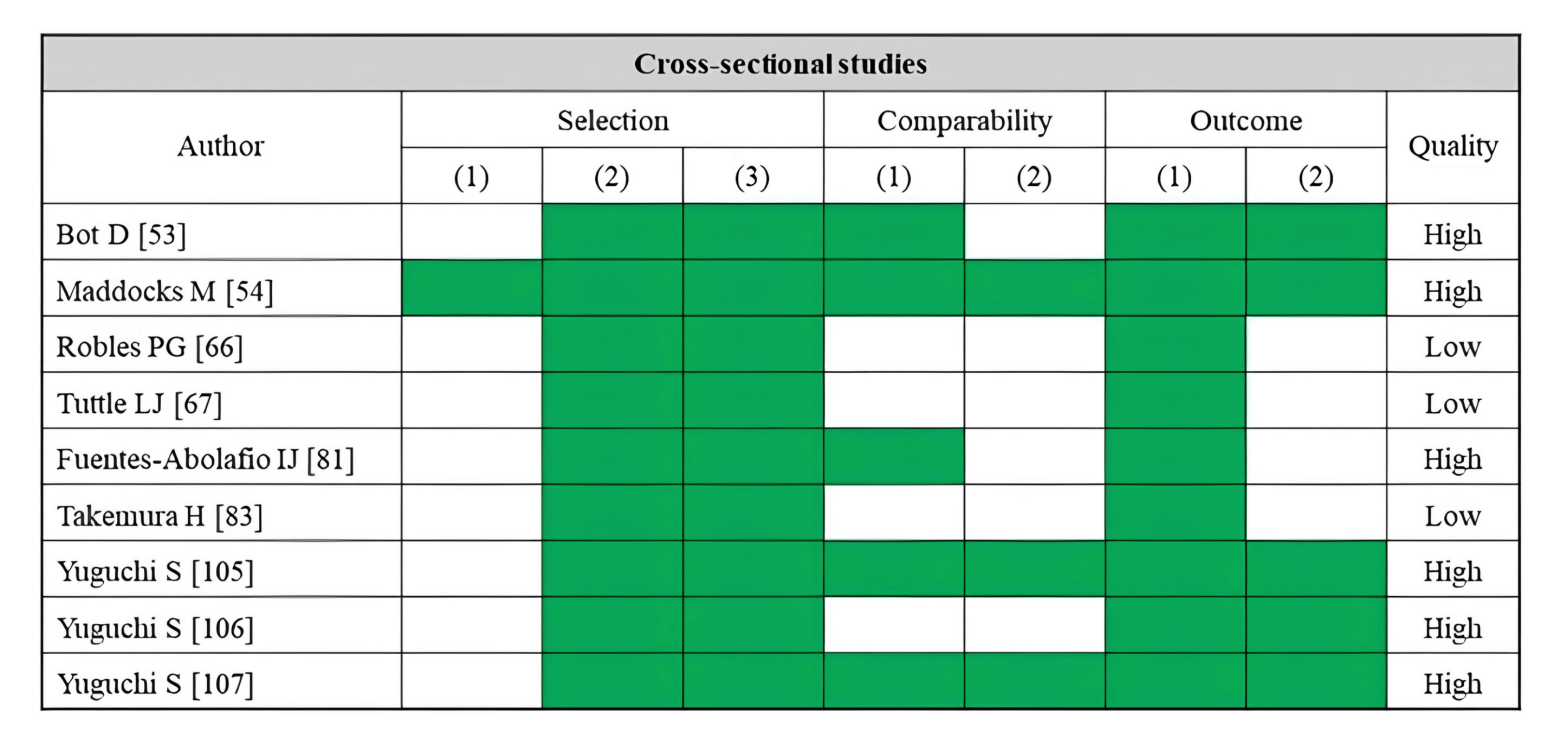
Assessment of risk of bias in studies on the 6MWT 6MWT, 6-min walk test

A total of nine studies reported 6MWT as an outcome (Table 8). Fatty infiltration of muscle was assessed by CT (two studies), MRI (two studies), and ultrasonography (five studies). The gastrocnemius was the most assessed skeletal muscle (three studies), followed by the rectus femoris (two studies). CT- and MRI-based studies consistently linked lower muscle density or greater adipose tissue in the trunk, thigh, and lower leg to shorter walking distance [53,54,66,67]. Ultrasonography-based studies consistently showed a correlation between higher echo intensity in the gastrocnemius and shorter walking distance [105-107]. Regarding the risk of bias assessment, six studies were rated as high quality, while three studies were rated as low quality (Figure 8).

**Table 8:** Association between fatty infiltration of muscle and the 6MWT 6MWT, 6-min walk test; β, standardized regression coefficient; COPD, chronic obstructive pulmonary disease; pQCT, peripheral quantitative CT

Author	Year	Study population, sample size (female, %), and age	Study design	Medical imaging modalities	Target muscle	Summary of the results
Bot D [53]	2023	Patients with liver disease, sample size: 130 (female: 36, 27.7%), age: 56.0 ± 11.0 years	Cross‐sectional	CT	Trunk muscles	Myosteatosis increased the odds of shorter walking distance (≤250 m) (OR: 3.857, 95% CI: 1.134–10.22).
Maddocks M [54]	2014	Patients with COPD, sample size: 101 (female: 41, 40.6%), age: 64.8 ± 7.6 years	Cross‐sectional	CT	Thigh muscles	Lower thigh muscle density was associated with shorter walking distance (β: 12.7, 95% CI: not reported).
Robles PG [66]	2015	Patients with COPD, sample size: 10 (female: 5, 50.0%), age: 70.1 ± 6.7 years	Cross‐sectional	MRI	Vastus lateralis and soleus	Higher lipid/total proton ratio in the vastus lateralis (r: −0.722) and soleus (r: −0.687) was associated with shorter walking distance.
Tuttle LJ [67]	2011	Patients with diabetes mellitus, sample size: 22 (female: 7, 31.8%), age: 64.5 ± 12.7 years	Cross‐sectional	MRI	Lower leg muscles	Greater adipose tissue in the lower leg was associated with shorter walking distance (r: −0.48).
Fuentes-Abolafio IJ [81]	2022	Patients with heart failure, sample size: 70 (female: 40, 57.1%), age: unclear	Cross‐sectional	Ultrasonography	Rectus femoris and vastus intermedius	No significant association was observed between the echo intensity of the rectus femoris and walking distance in men (r: −0.008) or women (r: 0.074).
Takemura H [83]	2022	Patients with COPD, sample size: 17 (female: 2, 11.8%), age: 83 (75–86) years	Cross‐sectional	Ultrasonography	Rectus femoris	No significant association was observed between the echo intensity of the rectus femoris and walking distance (r: −0.31).
Yuguchi S [105]	2023	Patients with peripheral arterial disease, sample size: 35 (female: 0, 0%), age: male, 77.0 ± 5.5 years; female, 73.7 ± 8.5 years	Cross‐sectional	Ultrasonography	Gastrocnemius	Higher echo intensity in the triceps surae was associated with shorter walking distance in both legs with high ankle-brachial index (ABI) (β: −0.46, 95% CI: not reported) and low ABI (β: −0.45, 95% CI: not reported).
Yuguchi S [106]	2022	Patients with peripheral arterial disease, sample size: 10 (female: 0, 0%), age (mean): 78.5 years	Cross‐sectional	Ultrasonography	Medial head of the gastrocnemius	Higher echo intensity in the medial head of the gastrocnemius was associated with shorter walking distance (r: −0.74).
Yuguchi S [107]	2022	Patients with peripheral arterial disease, sample size: 23 (female: 0, 0%), age: 74.5 ± 7.6 years	Cross‐sectional	Ultrasonography	Medial head of the gastrocnemius	In all models, higher echo intensity in the medial head of the gastrocnemius was associated with shorter walking distance (β: −0.74 to −0.71, 95% CI: not reported).

5STS

**Figure 9:**
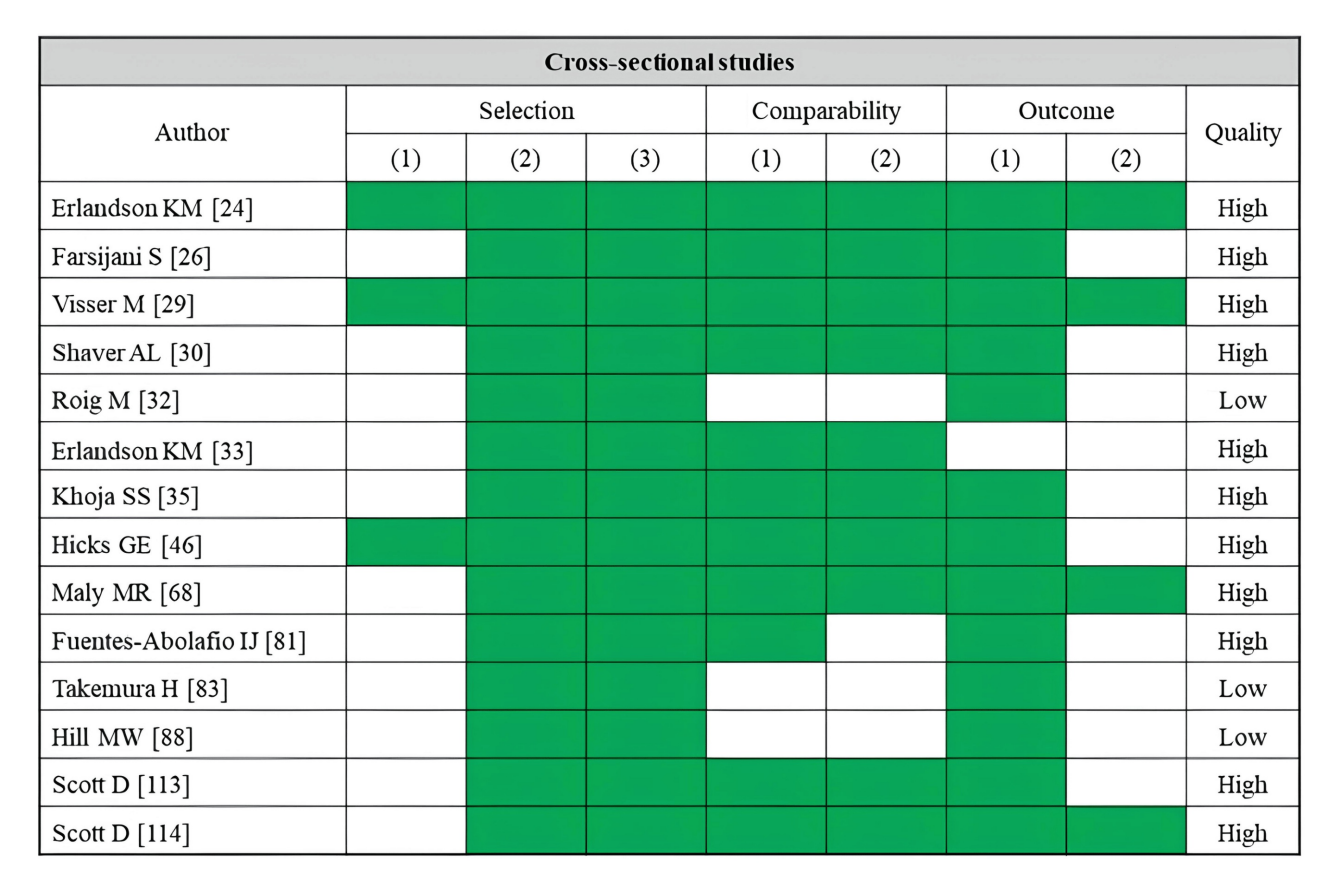
Assessment of risk of bias in studies on the 5STS 5STS, five times sit-to-stand test

A total of 14 studies reported 5STS as an outcome (Table 9). Fatty infiltration of muscle was assessed by CT (eight studies), MRI (nine study), ultrasonography (three studies), and pQCT (two studies). The whole thigh muscles were the most assessed skeletal muscle (six studies), followed by the rectus femoris (two studies), trunk muscle (two studies), and lower leg muscles (two studies). CT- and MRI-based studies reported inconsistent results regarding the relationship between muscle density or adipose tissue in the trunk and thighs and the time for 5STS [26,29,30,32,35,46,68]. No association was found between the echo intensity of the rectus femoris and the time on the 5STS [81,83]. However, pQCT-based studies consistently linked lower muscle density in the lower leg to prolonged time in the 5STS [113,114]. Regarding the risk of bias assessment, 11 studies were rated as high quality, while three studies were rated as low quality (Figure 9).

**Table 9:** Association between fatty infiltration of muscle and the 5STS β, standardized regression coefficient; 5STS, five times sit-to-stand test; COPD, chronic obstructive pulmonary disease; pQCT, peripheral quantitative CT

Author	Year	Study population, sample size (female, %), and age	Study design	Medical imaging modalities	Target muscle	Summary of the results
Erlandson KM [24]	2022	People infected with HIV, sample size: 571 (female: 184, 32.2%), age (range): 40–69 years	Cross‐sectional	CT	Rectus abdominis, anterolateral abdominal wall muscles, psoas major, paraspinal muscles, hamstrings, and quadriceps	In HIV-negative males, lower muscle density in the anterolateral abdominal wall (β: −0.2186), psoas major (β: −0.3142), paraspinal muscles (β: −0.2215), and quadriceps (β: −0.4259) was associated with prolonged time in the 5STS. In HIV-positive males, lower muscle density in the anterolateral abdominal wall (β: −0.1191), paraspinal muscles (β: −0.1267), and quadriceps (β: −0.3434) was associated with prolonged time in the 5STS. In females, no significant association was observed between muscle density and time in the 5STS.
Farsijani S [26]	2021	Community participants, sample size: 1897 (female: 991, 52.2%), age: 70 (73–85) years	Cross‐sectional	CT	Thigh muscles	Greater adipose tissue area in the thigh muscles was associated with prolonged time in the 5STS (β: −0.0014, 95% CI: not reported).
Visser M [29]	2002	Community participants, sample size: 2979 (female: 1537, 51.6%), age (range): 70–79 years	Cross‐sectional	CT	Thigh muscles	Lower muscle density in the thigh muscles was associated with prolonged time in the 5STS (White males, β: 0.156; Black males, β: 0.114; White females, β: 0.101; Black females, β: 0.008).
Shaver AL [30]	2022	Patients with cancer (head and neck), sample size: 90 (female: 19, 21.1%), age: 64.0 ± 10.7 years	Cross‐sectional	CT	Trunk muscles	No significant association was observed between trunk muscle density and time in the 5STS (overall, β: 0.37, 95% CI: −2.39–3.13; males, β: 0.20, 95% CI: −0.27–3.09; females, β: 8.62, 95% CI: −13.86–31.1).
Roig M [32]	2011	Patients with COPD, sample size: 21 (female: 10, 47.6%), age: 71.3 ± 8.1 years	Cross‐sectional	CT	Thigh muscles	No significant correlation was observed between the adipose tissue area in the thigh muscles and time in the 5STS (r: −0.43).
Erlandson KM [33]	2023	People infected with HIV, sample size: 139 (unclear), age: 51 (40–71) years	Cross‐sectional	CT	Paraspinal muscle	No significant association was observed between paraspinal muscle density and time in the 5STS (β: 0.665, 95% CI: −0.659–1.998).
Khoja SS [35]	2018	Patients with rheumatoid arthritis, sample size: 60 (female: 49, 81.7%), age: 59.0 ± 9.8 years	Cross‐sectional	CT	Thigh muscles	No significant association was observed between thigh muscle density and time in the 5STS (β: −0.004, 95% CI: not reported).
Hicks GE [46]	2005	Community participants, sample size: 1527 (female: 788, 51.6%), age: male, 73.9 ± 2.9 years; female: 73.5 ± 2.9 years	Cross‐sectional	CT	Trunk and thigh muscles	Lower muscle density in the trunk muscles was associated with prolonged time in the 5STS (β: 0.002, 95% CI: not reported). No significant association was observed between muscle density in the thigh muscles and prolonged time in the 5STS (β: 0.001, 95% CI: not significant).
Maly MR [68]	2013	Patients with knee osteoarthritis, sample size: 73 (female: 73, 100%), age: 64.6 ± 6.7 years	Cross‐sectional	MRI	Thigh muscles	Greater adipose tissue area in the thigh muscles was associated with prolonged time in the 5STS (β: 0.282, 95% CI: not reported).
Fuentes-Abolafio IJ [81]	2022	Patients with heart failure, sample size: 70 (female: 40, 57.1%), age: unclear	Cross‐sectional	Ultrasonography	Rectus femoris and vastus intermedius	No significant association was observed between the echo intensity of the rectus femoris and vastus intermedius and 5STS (males, r: 0.123; females, r: 0.074).
Takemura H [83]	2022	Patients with COPD, sample size: 17 (female: 2, 11.8%), age (mean): 83 years	Cross‐sectional	Ultrasonography	Rectus femoris	No significant association was observed between the echo intensity of the rectus femoris and 5STS (r: 0.44).
Hill MW [88]	2021	Community participants, sample size: 21 (female: 15, 42.9%), age: 69.9 ± 4.3 years	Cross‐sectional	Ultrasonography	Vastus lateralis and medial gastrocnemius	Higher echo intensity of the vastus lateralis (right leg: r, 0.568; left leg: r, 0.635) and medial gastrocnemius (right leg: r, 0.481; left leg: r, 0.59) was associated with prolonged time in the 5STS.
Scott D [113]	2018	Community participants, sample size: 48 (female: 25, 52.1%), age: 71.6 ± 4.8 years	Cross‐sectional	pQCT	Lower leg muscles	Lower muscle density in the lower leg was associated with prolonged time in the 5STS (β: −0.886, 95% CI: −1.553 to −0.179).
Scott D [114]	2015	Community participants, sample size: 48 (female: 25, 52.1%), age: 71.6 ± 4.8 years	Cross‐sectional	pQCT	Lower leg muscles	Lower muscle density in the lower leg was associated with prolonged time in the 5STS (β: −0.744, 95% CI: −1.259 to −0.228).

CS-30

**Figure 10:**
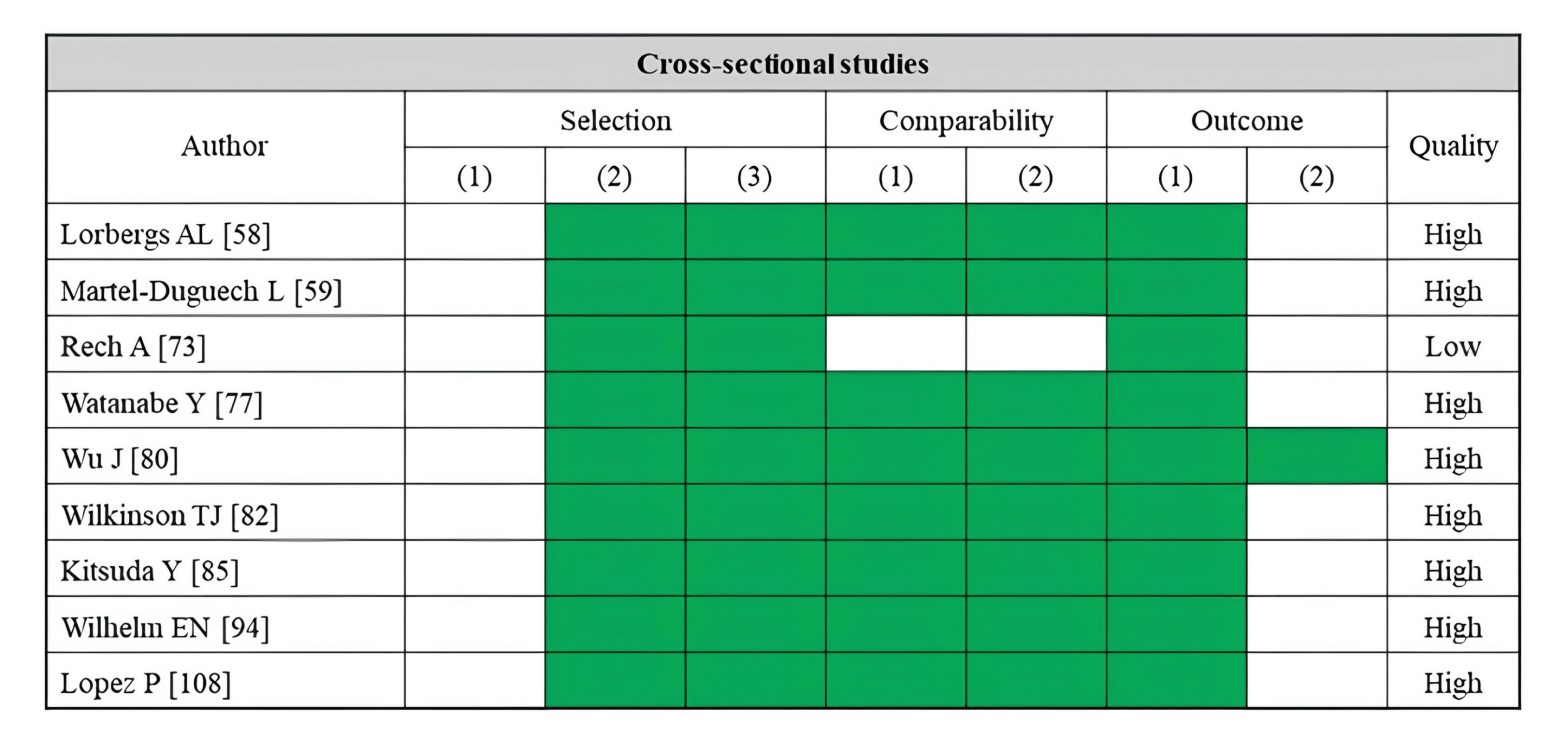
Assessment of risk of bias in studies on the CS-30 CS-30, 30-s chair stand test

A total of nine studies reported CS-30 as an outcome (Table 10). Fatty infiltration of muscle was assessed by MRI (2 studies) and ultrasonography (7 studies). Most of the skeletal muscle assessments were performed in the rectus femoris or quadriceps (4 studies each). MRI-based studies linked a higher percentage of adipose tissue area in the thigh to a reduced number of sit-to-stand repetitions [59], with no such association existing in the lower leg muscles [58]. Ultrasonography-based studies have uncovered a correlation between higher echo intensity in the quadriceps or rectus femoris and a reduced number of sit-to-stand repetitions [73,80,82,94,108]. Regarding the risk of bias assessment, 8 studies were rated as high quality, while 1 study was rated as low quality (Figure 10).

**Table 10:** Association between fatty infiltration of muscle and the CS-30 β, standardized regression coefficient; CS-30, 30-s chair stand test

Author	Year	Study population, sample size (female, %), and age	Study design	Medical imaging modalities	Target muscle	Summary of the results
Lorbergs AL [58]	2015	Community participants, sample size: 35 (female: 35, 100%), age: 70 (60–75) years	Cross‐sectional	MRI	Tibialis anterior, soleus, and gastrocnemius	No significant correlation was observed between the adipose tissue area of the tibialis anterior (r: −0.13), soleus (r: −0.31), or gastrocnemius (r: −0.08) and the number of sit-to-stand repetitions.
Martel-Duguech L [59]	2020	Patients with Cushing syndrome, sample size: 36 (female: 36, 100%), age: 51.0 ± 15.0 years	Cross‐sectional	MRI	Thigh muscles	Higher proportion of adipose tissue area in the thigh muscles was associated with reduced number of sit-to-stand repetitions (β: −0.524, 95% CI: not reported).
Rech A [73]	2014	Community participants, sample size: 45 (female: 45, 100%), age: 70.3 ± 6.2 years	Cross‐sectional	Ultrasonography	Quadriceps	Higher echo intensity in the quadriceps was associated with reduced number of sit-to-stand repetitions (r: −0.498).
Watanabe Y [77]	2019	Community participants, sample size: 53 (female: 28, 52.8%), age: male, 77.0 ± 5.5 years; female, 79.1 ± 6.1 years	Cross‐sectional	Ultrasonography	Quadriceps	No significant correlation was observed between the echo intensity of the quadriceps and the number of sit-to-stand repetitions (r: −0.120).
Wu J [80]	2022	Patients undergoing dialysis, sample size: 107 (female: 45, 37.4%), age: 53.5 ± 12.5 years	Cross‐sectional	Ultrasonography	Rectus femoris	Higher echo intensity in the rectus femoris was associated with reduced number of sit-to-stand repetitions (β: −0.081, 95% CI: not reported).
Wilkinson TJ [82]	2019	Patients with renal failure, sample size: 29 (female: 12, 41.4%), age: 57.5 ± 17.9 years	Cross‐sectional	Ultrasonography	Rectus femoris	Higher echo intensity in the rectus femoris was associated with reduced number of sit-to-stand repetitions (β: −0.356, 95% CI: −0.351 to −0.003).
Kitsuda Y [85]	2019	Patients who underwent total knee arthroplasty, sample size: 50 (female: 44, 88.0%), age: 76 (71−81) years	Cross‐sectional	Ultrasonography	Rectus femoris and vastus medialis	No significant association was observed between the echo intensity of the rectus femoris (β: −0.26, 95% CI: −0.76 to 0.24) or vastus medialis (β: −0.63, 95% CI: −1.27 to 0.03) and the number of sit-to-stand repetitions.
Wilhelm EN [94]	2014	Community participants, sample size: 50 (female: 0, 0%), age: 66.1 ± 4.5 years	Cross‐sectional	Ultrasonography	Quadriceps, rectus femoris, vastus intermedius, vastus medialis, and vastus lateralis	Higher echo intensities in the vastus lateralis (r: 0.413), rectus femoris (r: −0.296), vastus intermedius (r: −0.433), vastus medialis (r: −0.595), and the overall quadriceps (r: −0.502) were associated with reduced number of sit-to-stand repetitions.
Lopez P [108]	2017	Community participants, sample size: 50 (female: 0, 0%), age: 66.0 ± 5.4 years	Cross‐sectional	Ultrasonography	Quadriceps	Higher echo intensity in the quadriceps was associated with reduced number of sit-to-stand repetitions (β: −0.181, 95% CI: not reported).

Balance Test or Other Tests

**Figure 11:**
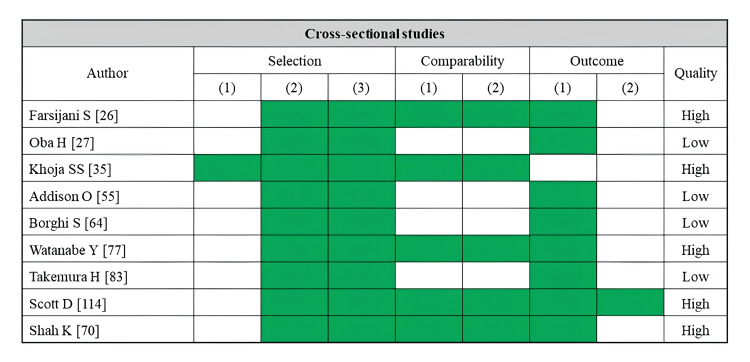
Assessment of risk of bias in studies on balance test or other tests

A total of eight studies reported the balance test as an outcome (Table 11). Fatty infiltration of muscle was assessed by CT (four studies), MRI (one study), ultrasonography (two studies), and pQCT (one study). The thigh muscles were the most assessed skeletal muscle (three studies), followed by the quadriceps (two studies). In addition, most studies have used single-leg standing time as an outcome. CT-based studies consistently linked lower muscle density or a higher proportion of adipose tissue in the thigh muscles or quadriceps to poorer performance on each balance test [26,27,35]. In addition, a greater adipose tissue area in the gluteus medius and gluteus minimus is associated with lower scores on the Berg Balance Scale and the dynamic gait index [55]. Although fatty infiltration of muscle has also been evaluated by MRI, ultrasonography, and pQCT, no significant association has been reported between it and any balance test [64,77,83,114]. Regarding risk of bias assessment, four studies were rated as high quality, while four studies were rated as low quality (Figure 11). Others reported performance tests that assess the ability to perform usual daily activities as an outcome [70]. Higher adipose tissue area in the thigh muscles of people infected with HIV was associated with lower scores on the physical performance test. The study was rated as high quality in terms of risk of bias.

**Table 11:** Association between fatty infiltration of the muscle and balance test or other tests β, standardized regression coefficient; COPD, chronic obstructive pulmonary disease; pQCT, peripheral quantitative CT; SPBB, short physical performance battery

Author	Year	Study population, sample size (female, %), and age	Study design	Medical imaging modalities	Target muscle	Summary of the results
Farsijani S [26]	2021	Community participants, sample size: 1897 (female: 991, 52.2%), age: 78 (73–85) years	Cross‐sectional	CT	Thigh muscles	Greater adipose tissue area in the thigh muscles was associated with the shorter total time for closed-stance standing, semi-tandem standing, and tandem standing (β: −0.2109, 95% CI: not reported).
Oba H [27]	2021	Community participants, sample size: 214 (female: 136, 63.5%), age (median): male, 78.3 years; female, 78.4 years	Cross‐sectional	CT	Quadriceps	Lower muscle density in the quadriceps was associated with shorter single-leg standing time (r: 0.24).
Khoja SS [35]	2018	Patients with rheumatoid arthritis, sample size: 60 (female: 49, 81.7%), age: 59.0 ± 9.8 years	Cross‐sectional	CT	Thigh muscles	Lower muscle density in the thigh was associated with shorter single-leg standing time (β: 0.416, 95% CI: not reported).
Addison O [55]	2014	Community participants, sample size: 48 (female: 28, 58.3%), age: 74.4 ± 1.0 years	Cross-sectional	CT	Psoas, gluteus maximus, gluteus medius and minimus, vastus lateralis, hamstrings, and the adductor magnus and longus	Greater adipose tissue area in the gluteus medius and minimus was associated with lower scores on the Berg Balance Scale (r: −0.36) and the dynamic gait index (r: −0.26).
Borghi S [64]	2022	Community participants, sample size: 34 (female: 24, 70.6%), age: 65.6 ± 4.7 years	Cross‐sectional	MRI	Thigh muscles	No significant correlation was observed between the adipose tissue area in the thigh muscles and the Mini-BESTest score (r: −0.132).
Shah K [70]	2020	Persons infected with HIV, sample size: 18 (female: unclear), age: 58.0 ± 5.0 years	Cross‐sectional	MRI	Thigh muscles	Higher adipose tissue area in the thigh muscles was associated with lower Physical Performance Test scores (r: −0.6).
Watanabe Y [77]	2019	Community participants, sample size: 53 (female: 28, 52.8%), age: male, 77.0 ± 5.5 years; female, 79.1 ± 6.1 years	Cross‐sectional	Ultrasonography	Rectus femoris	No significant correlation was observed between the echo intensity of the rectus femoris and either the single-leg standing time (r: 0.08) or the functional reach test (r: 0.317).
Takemura H [83]	2022	Patients with COPD, sample size: 17 (female: 2, 11.8%), age (mean): 83 years	Cross‐sectional	Ultrasonography	Rectus femoris	No significant correlation was observed between the echo intensity of the rectus femoris and the balance subscale of the SPBB (r: −0.4).
Scott D [114]	2015	Community participants, sample size: 48 (female: 25, 52.1%), age: 71.6 ± 4.8 years	Cross‐sectional	pQCT	Lower leg muscles	No significant association was observed between lower leg muscle density and Balance Outcome Measure for Elder Rehabilitation scores (β: 4.726, 95% CI: −0.930–10.382).

Discussion

Our systematic review integrated 97 observational studies to comprehensively examine the association between fatty infiltration of muscle, falls, and fall-related outcomes. As a result, a number of studies have shown that fatty infiltration of muscle may be associated with falls. Similarly, fatty infiltration of muscle has been shown to be associated with a poorer variety of fall-related outcomes. On the other hand, the studies included in our systematic review varied in participant characteristics, and methods for assessing fatty infiltration of muscle, and exhibited a mixed risk of bias. Thus, our systematic review provides important evidence on assessing fatty infiltration of muscle for fall prevention in older adults, while underscoring the need for careful interpretation and further research, considering variations in participant characteristics, assessment methods, and potential biases.

The novelty of our systematic review is that it systematically organized the direct influence of fatty infiltration of muscle on falls and fall-related outcomes. Previous systematic reviews have consistently shown that sarcopenia and muscle weakness are important predictors of falls [20,117]. However, the studies included in the review focused on muscle mass and strength, and the effect of morphological changes in muscle on falls was not systematically investigated. Our systematic review focuses on fatty infiltration of muscle as an indicator of morphological changes in muscle and comprehensively examines its relationship not only with falls but also with various fall-related outcomes. Therefore, this systematic review highlights the importance of evaluating fatty infiltration of muscle when considering fall prevention measures and may provide a new perspective for future research and clinical practice.

Our review suggests that fatty infiltration of the lower extremity muscles is a risk factor for falls. Fatty infiltration of muscle reduces muscle function by increasing the amount of noncontractile tissue within the muscle, selectively disrupting type 2 fibers and impairing neuromuscular control [118]. In addition, fatty infiltration of the lower extremity muscles may be associated with increased gait variability [55] and an inability to implement appropriate balancing strategies during external perturbations [119]. These previous results support our review findings regarding Fatty infiltration of muscle and falls. Thus, fatty infiltration of the lower extremity muscles may interfere with normal muscle function and increase fall risk by decreasing stability during gait and the ability to respond to high balance demands.

Our review results also suggest that fatty infiltration of muscles affects various fall-related outcomes, among which comfortable walking speed, TUG, and SPPB are frequently linked to fatty infiltration of muscles. These outcomes are important indicators for identifying patients at high risk of falls in fall prevention guidelines [120]. Interestingly, many of these outcomes were also associated with the fatty infiltration of the trunk muscles, not just the lower extremity muscles. Trunk muscles play an important role in postural control in antigravity positions [121]. They are also highly correlated with total body muscle mass [122], which may reflect physical frailty. Therefore, understanding trunk fatty infiltration of the trunk muscles may help prevent the deterioration of functional status, which increases fall risk.

Despite the potential influence of fatty infiltration of muscle on fall risk, some studies have reported no association. This suggests that fatty infiltration of muscle assessment methods can be influential. Most of the currently reviewed studies relate to fatty infiltration of the rectus femoris and quadriceps. The association between fat infiltration and various outcomes in these muscles varied based on the assessment method (echo intensity vs. muscle density or fat area), with many studies reporting no association when echo intensity was used. Scoping reviews of muscle quality assessment report that echo intensity is the most used indicator [12]. However, technical factors - such as probe angle and pressure during measurement - can affect echo intensity, potentially influencing the results [123]. In addition, the coefficient of variation of echo intensity exceeds that of measurements obtained using other medical imaging modalities [123,124]. Thus, the current study highlights the variability in these fatty infiltrations of muscle measures and suggests the best assessment method. This will contribute to the future standardization of fatty infiltration of muscle assessment methods.

Interventions targeting local and systemic fatty infiltration of muscle to prevent falls should be considered. Fatty infiltration of muscle, the focus of our review, is a modifiable factor [125-128]. Resistance training [125] and neuromuscular electrical stimulation [127] can alleviate fatty infiltration of muscle, which may be useful for localized muscles in the lower extremities. In addition, the effectiveness of aerobic exercise training [127] and nutritional supplementation interventions [128] on fatty infiltration of muscle has been demonstrated. These methods may be an option if the goal is to improve systemic fatty infiltration of muscle. Therefore, a multifaceted intervention strategy that addresses fatty infiltration of muscle may be an effective approach for preventing falls and mitigating physical function deterioration. Nevertheless, this hypothesis requires further corroboration.

Our systematic review has some limitations. First, this review included only observational studies, the majority of which were cross-sectional. Thus, directly establishing a causal relationship between fatty infiltration of muscle and falls or fall-related outcomes is difficult. Second, no meta-analysis was performed in this review. Different methods were used to assess fatty infiltration of muscle, including CT, MRI, ultrasonography, and pQCT. The measurement indices or analysis methods used lacked uniformity. In addition, the study populations varied widely, including community-dwelling older adults, hospitalized patients, and populations with various medical conditions. This results in high heterogeneity between the studies. As most included studies focused on the fatty infiltration of a single muscle, determining the relative contribution of each muscle to falls and fall-related outcomes remains impossible. Thus, the extent to which specific muscles contribute to fall risk or fall-related outcomes remains undetermined. Third, most included studies were at high risk of bias in terms of self-selection bias and comparability (Figure 2, Figure 3, Figure 4, Figure 5, Figure 6, Figure 7, Figure 8, Figure 9, Figure 10, Figure 11). These biases suggest concerns regarding the generalizability and independence of the fatty infiltration of muscle influence on fall risk. Finally, only studies published in English and Japanese were included, which may have introduced relevant language biases and limited the generalizability of our findings. Despite these limitations, this review is the first report, to the best of our knowledge, to consolidate evidence of the association between fatty infiltration of muscle and falls or fall-related outcomes. Further development of intervention and longitudinal studies is expected to further clarify the effectiveness of fall prevention strategies that utilize fatty infiltration of muscle assessments.

## Conclusions

Our systematic review suggests that fatty infiltration of the muscle may be associated with an increased risk of falls and poor fall-related outcomes in middle-aged and older adults. However, the current evidence is limited by methodological heterogeneity and potential biases, underscoring the need for further well-designed studies to confirm these associations and inform clinical practice.
